# Identifying Cognate Binding Pairs among a Large Set of Paralogs: The Case of PE/PPE Proteins of *Mycobacterium tuberculosis*

**DOI:** 10.1371/journal.pcbi.1000174

**Published:** 2008-09-12

**Authors:** Robert Riley, Matteo Pellegrini, David Eisenberg

**Affiliations:** 1 Department of Human Genetics, David Geffen School of Medicine, University of California Los Angeles, Los Angeles, California, United States of America; 2 Howard Hughes Medical Institute, University of California Los Angeles, Los Angeles, California, United States of America; 3 UCLA–DOE Institute of Genomics and Proteomics, University of California Los Angeles, Los Angeles, California, United States of America; 4 Genome Biology Program, Broad Institute of MIT and Harvard, Cambridge, Massachusetts, United States of America; 5 Department of Molecular, Cell, and Developmental Biology, University of California Los Angeles, Los Angeles, California, United States of America; Max-Planck-Institut für Informatik, Germany

## Abstract

We consider the problem of how to detect cognate pairs of proteins that bind when each belongs to a large family of paralogs. To illustrate the problem, we have undertaken a genomewide analysis of interactions of members of the PE and PPE protein families of *Mycobacterium tuberculosis*. Our computational method uses structural information, operon organization, and protein coevolution to infer the interaction of PE and PPE proteins. Some 289 PE/PPE complexes were predicted out of a possible 5,590 PE/PPE pairs genomewide. Thirty-five of these predicted complexes were also found to have correlated mRNA expression, providing additional evidence for these interactions. We show that our method is applicable to other protein families, by analyzing interactions of the Esx family of proteins. Our resulting set of predictions is a starting point for genomewide experimental interaction screens of the PE and PPE families, and our method may be generally useful for detecting interactions of proteins within families having many paralogs.

## Author Summary

We consider the problem of detecting protein interactions from genome sequences when the potential interacting partners belong to large families of similar (homologous) proteins. Many computational methods for predicting protein interactions rely on similarity to a pair of known interacting proteins. When the proteins in question are members of large groups of similar proteins within the same organism (paralogs), the problem of inferring the correct interactions becomes difficult. To illustrate the problem, we undertook prediction of interactions of some highly expanded protein families of *Mycobacterium tuberculosis* (*Mtb*), which are believed to contribute to the bacterium's ability to infect human beings. To generate predictions, we analyzed patterns of coevolution in a small subset of likely interacting proteins, and extended these patterns to predict additional interactions throughout the genome. Our results provide a map for experimental probes of the *Mtb* interaction network, for the benefit of drug and vaccine discovery. More generally, our procedure is applicable to detecting interactions of proteins that belong to large families of paralogs in any organism with a sequenced genome.

## Introduction

Tuberculosis remains a health problem of global importance [1]. Despite the availability of the genome sequence of *Mycobacterium tuberculosis* (*Mtb*) for nearly a decade [2], the biology of the pathogen, particularly the molecular mechanisms by which it achieves virulence, remains poorly understood. Probing the molecular interaction network of *Mtb* therefore is an important step in the fight against tuberculosis disease.

### The PE and PPE Families

The PE and PPE gene families in *Mtb* make up nearly 10% of the bacterium's coding DNA [2]. The two families combined have about 150 members, amounting to 4% of the open reading frames (ORFs) in *Mtb*. The PE and PPE gene families account for much of the genomic difference between *Mtb* and other (nonpathogenic) mycobacterial genomes [3],[4]. Therefore it is thought that they may have a role in *Mtb*'s virulence and host-specificity. A subset of PE proteins is displayed on the bacterium's cell surface [5], can elicit an immune response [6], and may be a source of antigenic diversity for *Mtb* [7]. PPE proteins have also been found on the cell surface [8],[9], may be secreted [10], and can confer virulence [11]. These studies indicate the likely importance of the PE and PPE gene families in pathogenesis. More extensive characterization of their function, interactions, and roles in infection are therefore important areas for investigation.

Genome analysis suggests that the PE and PPE families are functionally linked [12]–[14]. Pairs of PE and PPE genes are frequently found adjacent on the *Mtb* genome, and the structure of a complex of one such PE/PPE protein pair was recently characterized [13]. These results indicate that there may be many other instances of interactions between PE and PPE proteins. However, with only one complex characterized so far, it remains unclear which specific members of the two families interact. The 87 PE and 65 PPE proteins (depending on similarity threshold) in the *Mtb* H37Rv genome generate ∼6,000 possible pairwise combinations. It may be that dozens of biologically relevant PE/PPE complexes remain to be characterized. Because the PE and PPE families can interact with the host immune system [5],[6],[11], combinatorial formation of complexes might enable immune evasion during tuberculosis infection. Mapping the PE/PPE interaction network is therefore of critical importance for accelerating drug discovery. Because PE and PPE proteins are difficult to express and purify experimentally [13], new computational methods are needed to detect likely PE/PPE complexes and efficiently prioritize experiments.

### Detection of Interacting PE and PPE Proteins

Perhaps the most straightforward bioinformatic approach for detecting PE/PPE complexes is to simply predict interaction of the PE/PPE pairs found in the same operon [15]–[18]. Some 14 pairs of PE and PPE genes, including the one complex that has been structurally characterized to date [13], are found adjacent on the genome, in the same orientation, with minimal intergenic distance, and with the PE 5′ to (upstream of) the PPE (the PE proteins in such pairs do not include any of the repeat-containing PE_PGRS proteins). Because of this recurring genome organization motif, such pairs are likely expressed in the same operon [19]. However, these same-operon PE/PPE pairs comprise less than 10% of the total number of PE and PPE genes in *Mtb*. The majority of PE and PPE proteins are found unpaired in the genome, and it is possible that some of these interact despite not having genomic proximity. Computationally detecting PE/PPE complexes not found by the operon method is therefore an important challenge.

The tendency for proteins to coevolve with their interaction partners has been described [20]–[23], and bioinformatic methods to detect protein coevolution have therefore been proposed for predicting protein interactions [24]. The idea is to exploit the correlation of the phylogenetic distance matrices of two protein families whose members are known from experiments to interact. Known interacting proteins tend to be found at analogous regions of their respective phylogenetic trees [20],[24],[25] (which can also be represented as distance matrices). Such methods can accurately pair ligands with receptors [24], and could potentially be used to infer interactions between the PE and PPE families. However, a difficulty of applying these methods in our case is that benchmarking predictions requires a set of experimentally determined interactions, and currently only a single known example of a PE/PPE complex exists [13]. Our challenge, therefore, for the computational prediction of PE/PPE interactions is the evaluation of predictions given the currently limited number of known PE/PPE interactions.

We combined the operon method, coevolution analysis, and structural knowledge of interacting domains to develop a coevolution-based strategy to predict PE/PPE complexes in the *Mtb* H37Rv strain. Some 289 predicted complexes resulted from the application of our method. To validate the predictions, we used several published mRNA expression datasets from *Mtb* to assess PE/PPE coexpression in vivo. A significant overlap was seen between coevolved and coexpressed PE/PPE gene pairs, supporting the coevolution-based predictions, and resulting in a high-confidence list of possible complexes. To demonstrate the extensibility of our method to other protein families, we performed a similar analysis of interactions of the ESAT-6/CFP-10 (Esx) family of proteins. Our results are a starting point for experimental genomewide screens of PE/PPE and Esx complexes, and our method may be applicable to other functionally linked protein families in *Mtb* and other microbial pathogens.

## Results

### Assumptions

We assumed that each interacting pair of PE/PPE proteins must have complementary interfaces, and that the residues in these interfaces may coevolve due to positive selective pressure on the interaction. Although we currently do not have sufficient data from PE/PPE complexes to accurately predict residue-residue interactions from sequence using correlated mutations analysis [26]–[29], we can delineate the likely interacting regions by their similarity to the structurally characterized PE/PPE interacting domains [13].

**Figure 1:**
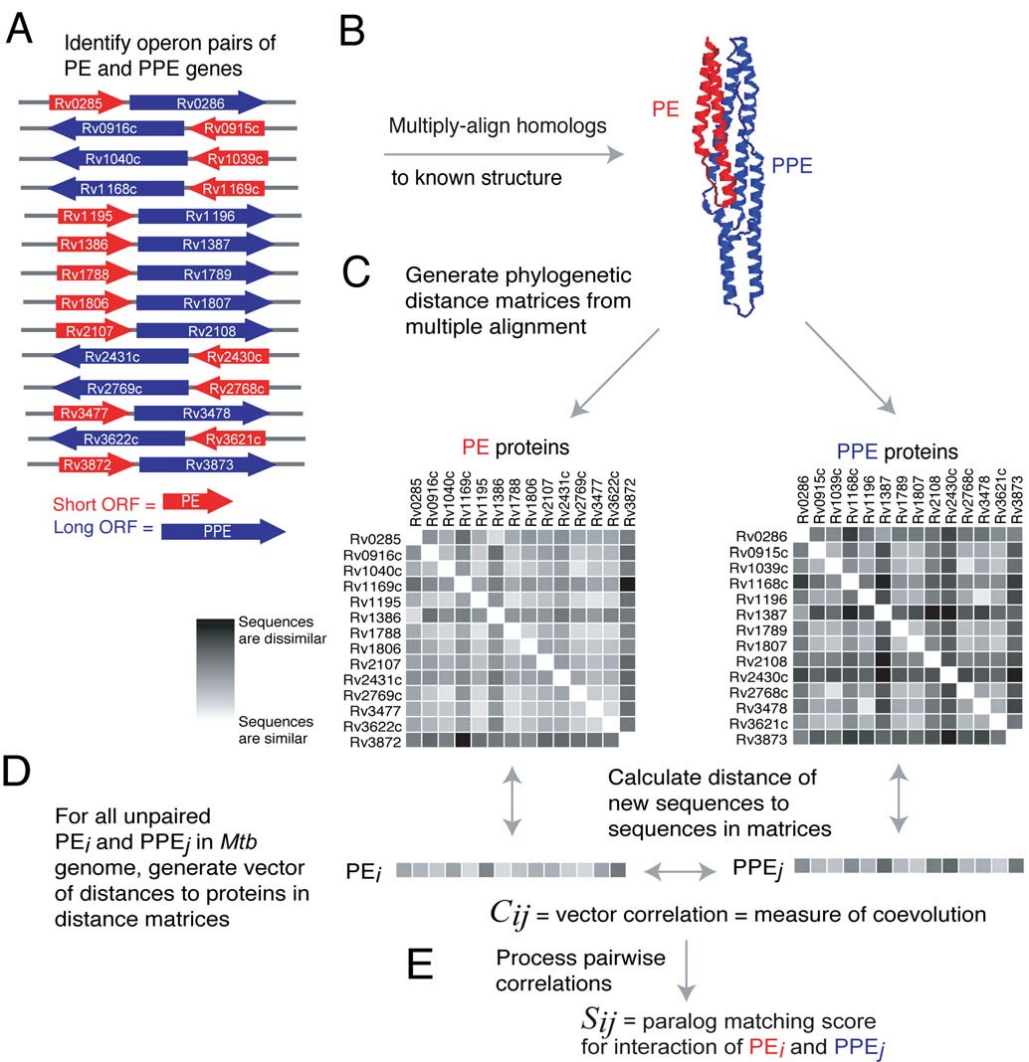
Overview of method for prediction of PE/PPE complexes. (A) PE/PPE operon pairs are identified. (B) Protein sequences of PE/PPE operon pairs are aligned to the known PE/PPE structure [13]. (C) Phylogenetic distance matrices for operon pairs are generated from the multiple alignments. (D) Coevolution of all genomewide PE/PPE pairs is evaluated by comparing distance vectors of length 14, consisting of the sequence distances between each protein and its 14 homologs in the PE or PPE reference matrix. (E) Coevolution correlations are further processed to generate predicted PE/PPE complexes.

We assumed that PE/PPE gene pairs adjacent on the genome, and in the same orientation, are in expression operons, as has been shown for Rv2431c/Rv2430c [13]. The components of protein complexes and metabolic pathways in prokaryotes are often located together on the genome in operons [19]. These operons are transcribed as a single, polycistronic mRNA. Genes located on an operon usually function together, and often form protein complexes. We predict thirteen other PE/PPE gene pairs lie in operons (Figure 1A) based on their short intergenic distance (<100 bp) and same transcription direction. These pairs have a high degree of coexpression (average mRNA correlation 0.59 for operon-paired, 0.05 for genomewide PE/PPE gene pairs, see Materials and methods), suggesting that these PE/PPE pairs are indeed in operons.

Finally, we assumed that PE/PPE pairs in operons are likely to interact in a manner similar to the structurally characterized, operon-coded, PE/PPE complex of Rv2431c/Rv2430c [13]. To support our assumption that bacterial operons tend to code protein complexes, we analyzed the tendency for annotated *E. coli* protein complexes to reside in operons in the EcoCyc database [30]. We extracted 280 complexes, involving 692 proteins, from EcoCyc. We asked what fraction of protein pairs found in complexes also had their genes in the same operon, and found this to be 49% (942 protein pairs in operons out of 1918 protein pairs in complexes). To assess the significance of this result, we shuffled the identity of the genes in complexes by replacing each with a random *E. coli* gene, and re-assessing the overlap. One thousand shufflings were performed and an overlap of 49% was never achieved; in fact, the highest overlap obtained was 2%. We conclude there is a significant tendency for bacterial protein complexes to be coded in the same operon. While this does not guarantee that proteins coded in operons interact, given a known example of an operon-coded PE/PPE complex, we might expect PE/PPE pairs similarly organized in operons to interact.

### Schematic Explanation of Our Method

Figure 1 illustrates our method for detecting pairs of coevolved PE and PPE genes (and thus, possible interacting proteins). Figure 1A shows all PE and PPE gene pairs that lie in the same orientation of 5′ PE → PPE 3′ with no more than 100 bp separation between the PE and PPE genes. These PE/PPE pairs are likely within the same operon [15]–[18], and are summarized in Table 1. We refer to these as the ‘operon pairs’; they form the training data for our method. PE and PPE protein sequences coded by the operon pairs are aligned to the sequence of the appropriate subunit of the PE/PPE complex of Rv2431c/Rv2430c (Figure 1B). Next, the structure-based multiple alignment is used to generate phylogenetic distance matrices, which contain pairwise protein similarity relationships (Figure 1C). Notice that each equivalent row in the matrix is an operon-paired (and hence assumed interacting) PE/PPE pair. These are called the ‘reference matrices’. For all (operon-paired or otherwise) PE and PPE protein sequences in the *Mtb* genome, distance vectors to the reference matrices are generated (Figure 1D). The correlation between these vectors, *C_ij_*, is a measure of the PE/PPE pair's possible coevolution. Next, *C_ij_* scores are further processed (Figure 1E) to yield *S_ij_*, the paralog matching score for the predicted complex of PE*_i_* and PPE*_j_*.

**Table 1:** The 14 pairs of PE and PPE genes linked by the operon method.

PE ORF	PE name	Length (aa)	Strand	PPE ORF	PPE name	Length (aa)	Strand	Genomic distance (bp)
Rv0285	PE5	102	+	Rv0286	PPE4	513	+	3
Rv0916c	PE7	99	−	Rv0915c	PPE14	423	−	15
Rv1040c	PE8	275	−	Rv1039c	PPE15	391	−	77
Rv1169c	PE11	100	−	Rv1168c	PPE17	346	−	18
Rv1195	PE13	99	+	Rv1196	PPE18	391	+	47
Rv1386	PE15	102	+	Rv1387	PPE20	539	+	3
Rv1788	PE18	99	+	Rv1789	PPE26	393	+	14
Rv1806	PE20	99	+	Rv1807	PPE31	399	+	27
Rv2107	PE22	98	+	Rv2108	PPE36	243	+	56
Rv2431c	PE25	99	−	Rv2430c	PPE41	194	−	47
Rv2769c	PE27	275	−	Rv2768c	PPE43	394	−	80
Rv3477	PE31	98	+	Rv3478	PPE60	393	+	37
Rv3622c	PE32	99	−	Rv3621c	PPE65	413	−	10
Rv3872	PE35	99	+	Rv3873	PPE68	368	+	31

These pairs of PE and PPE genes are unique in that they are oriented with the PE protein 5′ (upstream) to the PPE protein, and with no more than 100 base pairs intergenic separation between them. We refer to these as ‘operon pairs’; they make up the training data for our method.

The probable interacting regions of all PE and PPE proteins in the *Mtb* genome were delineated. This was done using the ClustalW program [31] to perform a multiple sequence alignment of each protein family to a secondary structure profile derived using the DSSP program [32] on the appropriate subunit of the known PE/PPE complex structure. The secondary structural alignment was motivated by the observation that the PE and PPE proteins of known structure are composed of long α-helices interspersed with turns and loops, and our intuition that insertions and deletions would preferentially occur in regions outside the helices. The alignment was visually inspected to remove outlying or poorly-aligned sequences. All remaining PE and PPE sequences in the alignment were truncated to eliminate regions not aligned to the structure. In many cases both the PE and PPE proteins contained additional domains in their C-terminal regions, including the PGRS repeats in PE_PGRS proteins. All subsequent sequence analysis in this work was performed with these truncated sequences. We reasoned that limiting our analysis to homologous interacting domains would facilitate detection of coevolution relevant to protein interaction, and would therefore not be confused by spurious coevolution signals from regions not involved in PE/PPE interface. This additional truncation step was performed because we observed that most PPE, and some PE, domains have additional domains, low complexity regions, or membrane helices C-terminal to the conserved interacting domains. The resulting alignments are provided in the Supporting Information ( Datasets S1 and S2).

### Phylogenetic Distances of PE/PPE Pairs

Phylogenetic distance matrices for the subset of PE and PPE proteins linked in the 14 operons (Table 1) were constructed using the ClustalW program [31]. For each of the PE and PPE families, the 14 sequences in operon pairs were manually extracted from the full-family alignment. The 14-sequence subalignments were then loaded into ClustalW to generate 14×14 distance matrices. Phylogenetic distance matrices represent the pairwise distance between protein sequences. In ClustalW, pairwise distances between sequences are measured by the fraction of mismatches in ungapped positions of an alignment of two sequences. If our assumption that operon-paired PE/PPE genes code complexes were correct, we reasoned that there would be a correlation of the two distance matrices when the genes of both matrices are respectively ordered by the genomic position of the operon in which they occur (Figure 1C). Such a correlation between matrices would be consistent with previous analyses demonstrating correlation of distance matrices for known interacting proteins [20]. We indeed found that the PE and PPE matrices were correlated, with a Pearson correlation coefficient of 0.84.

To assess the significance of the correlation of the PE and PPE matrices, we performed random shuffling of the matrices' gene order, thus removing any mapping of paired genomic position between the matrices. One million shuffling steps were performed, and the frequency with which the shuffled correlation exceeded the correlation from the operon-ordered matrices was recorded. The correlation of 0.84 was never exceeded in 10^6^ matrix shuffling steps (the maximum correlation in any shuffling was 0.20). These results suggest that the PE and PPE matrices, ordered by operon position, may represent an optimal pairing of PE/PPE proteins, and, in light of previous findings of correlated distance matrices of interacting proteins [20], support the hypothesis that PE/PPE operons code complexes.

**Figure 2:**
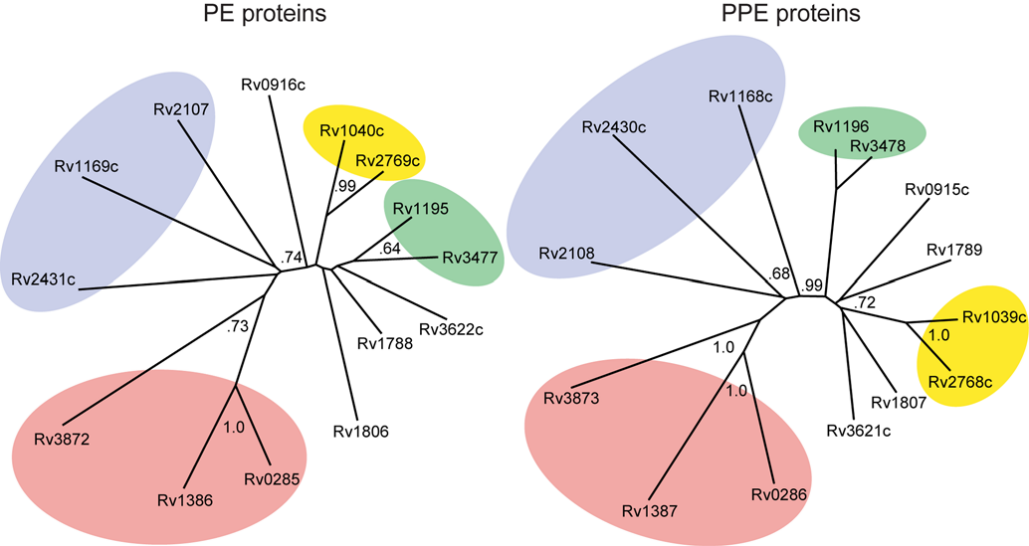
Phylogenetic tree of operon-paired PE and PPE proteins. Regions shaded the same color contain proteins from the same operons, indicating similar topologies of the trees. Bootstrap values >0.50 are shown in gray. This qualitative tree similarity illustrates the notion of coevolution in interacting protein families.

The correlation of the operon-ordered distance matrices can be visualized using phylogenetic trees to provide an intuitive feeling for the results. To illustrate this, we generated trees from distance matrices in the ClustalW program (Figure 2). In the two trees, operon-paired PE and PPE proteins are in the same-colored shaded region, illustrating similar topologies of the trees. This qualitative tree similarity illustrates the notion of coevolution of the PE and PPE families.

### Predictive Scores

We next used the correlated 14×14 PE and PPE distance matrices as reference matrices to evaluate pairwise correlations between the 86 PE proteins and 65 PPE proteins in the *Mtb* genome, excluding those present in operon pairs. This was done by generating a distance vector of length 14 for each protein in a PE/PPE pair. The vector contained the distance between the protein being tested and the 14 members of the appropriate reference matrix (PE or PPE). The Pearson correlation for the two vectors was calculated to obtain a measure of the coevolution of the test PE/PPE pair (Figure 1E). Notice that here we are taking the correlation of two vectors of length 14, as opposed to our earlier calculation of the correlation of the two 14×14 matrices. The coevolution of 5590 PE/PPE pairs was evaluated using this approach. We define the coefficient *C_ij_*, as the correlation which measures the coevolution of PE*_i_* with PPE*_j_*.

**Figure 3:**
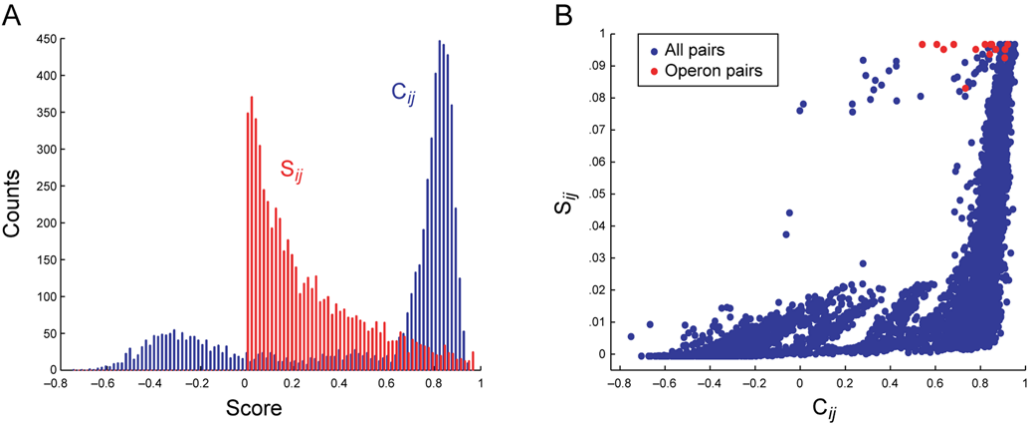
Predictive score distributions. (A) Score histogram of *C_ij_* and *S_ij_*. Notice that *C_ij_* has a high density of scores near the maximum value, making it unsuitable for protein interaction prediction, whereas *S_ij_* gives lower scores to most PE/PPE pairs, and higher scores to relatively few. (B) *S_ij_* plotted against *C_ij_*. PE/PPE operon pairs are shown in red. Notice that *S_ij_* preferentially assigns high scores to the operon pairs compared to *C_ij_*.

Pairwise correlations between all PE*_i_* and PPE*_j_* (*C_ij_*) were further processed using a reciprocal ranking procedure, to produce a predictive paralog matching score, *S_ij_*. This was done because we noticed that many PE/PPE pairs had high *C_ij_* values (average *C_ij_* = 0.54). The distribution of *C_ij_* is shown in Figure 3A (blue bars). From the histogram, it is clear that a great number of PE/PPE pairs have a high *C_ij_*. This may reflect the overall coevolution of the two families, but is not of use in a prediction scheme, as nearly all pairs have a high score. Such a result is inconsistent with our intuition that in a large collection of proteins, only a relatively small number of the possible pairs should interact. Further, we found that the *C_ij_* distributions for operon pairs and all PE/PPE pairs do not differ significantly (Kolmogorov–Smirnoff (KS) test, *α* = 0.05, *k* = 0.29, *P* = 0.16). In the reciprocal ranking procedure, the predicted complex of PE*_i_* and PPE*_j_* was assigned a high *S_ij_* only if PE*_i_* and PPE*_j_* were mutually at the top of each protein's list of interaction partners ranked by *C_ij_*. In other words, PE*_i_* and PPE*_j_* were required to be reciprocally the most coevolved partners in order to get a high *S_ij_* (see Materials and methods). Figure 3A, shows the distribution of *S_ij_* scores (red bars). The distribution of *S_ij_* suggests it is a more useful measure than *C_ij_* for complex prediction, as the bulk of the *S_ij_* scores are low (reflecting that in a large dataset, most protein pairs do not form complexes). The operon pairs have a significantly higher *S_ij_* than PE/PPE pairs overall (KS test, *α* = 0.05, *k* = 0.97, *P*–value<.0001), a result illustrated in Figure 3B. We conclude that the reciprocally ranked coevolution score, *S_ij_*, performs better than *C_ij_* for predicting protein interactions.

### Evaluation of Predictive Scores

To evaluate the predictive value of the two pairwise PE/PPE scores, *C_ij_* and *S_ij_*, we assessed recovery of the 14 operon linked PE/PPE pairs used to generate the reference matrices. Of the 14 pairs, all were given *S_ij_* scores in the top 5% implying the method could be used to detect complexes with reasonably high accuracy. In contrast, only 3/14 (20%) of the operon pairs were given *C_ij_* scores in the top 5%. A mere 8/14 pairs (60%) had *C_ij_* scores above the median, implying poor recovery by the raw distance vector correlations. The relationship between *C_ij_* and *S_ij_* is illustrated in Figure 3B. The distribution of scores shows that *S_ij_* as a predictor of PE/PPE complexes gives much better recovery of operon-paired PE/PPE proteins than *C_ij_*, and therefore is likely a better indicator of interaction.

To further evaluate prediction accuracy, and to determine a prediction score threshold, we compared the sensitivity (also called true positive rate or TPR) and 1-specificity (also called the false positive rate or FPR) for *S_ij_* and *C_ij_*. Sets of positive and negative interactions were defined, and an *S_ij_* threshold of 0.75 was found to capture the best balance between sensitivity and specificity (Materials and methods). Applying a prediction criterion of *S_ij_*≥0.75 gave 289 PE/PPE pairs or roughly 5% of the possible 5,590 PE/PPE pairs genomewide. We therefore proceeded with our analysis by taking the predictions with *S_ij_* scores in the top 5%.

### Correlation of mRNA Expression

To see if the coevolution-based predictions were biologically sensible, we analyzed correlations in mRNA expression (which we call ‘coexpression’) of possible interacting PE/PPE pairs. We reasoned that interacting proteins would tend to be expressed at similar times to perform their biological functions together [33]–[37], and that if some of our predicted interactions were correct, we should see non-random enrichment in coexpression of the genes encoding the predicted complexes. Gene microarray data from *Mtb* was compiled from nine published datasets covering a broad range of experimental conditions (Table S1) in the Gene Expression Omnibus (GEO) database [38]. Vectors of expression values for each PE or PPE gene were generated, and used to derive a correlation, *R_ij_*, for the expression of each PE/PPE gene pair (see Materials and methods).

### Overlap of Results from Coexpression and Coevolution

**Figure 4:**
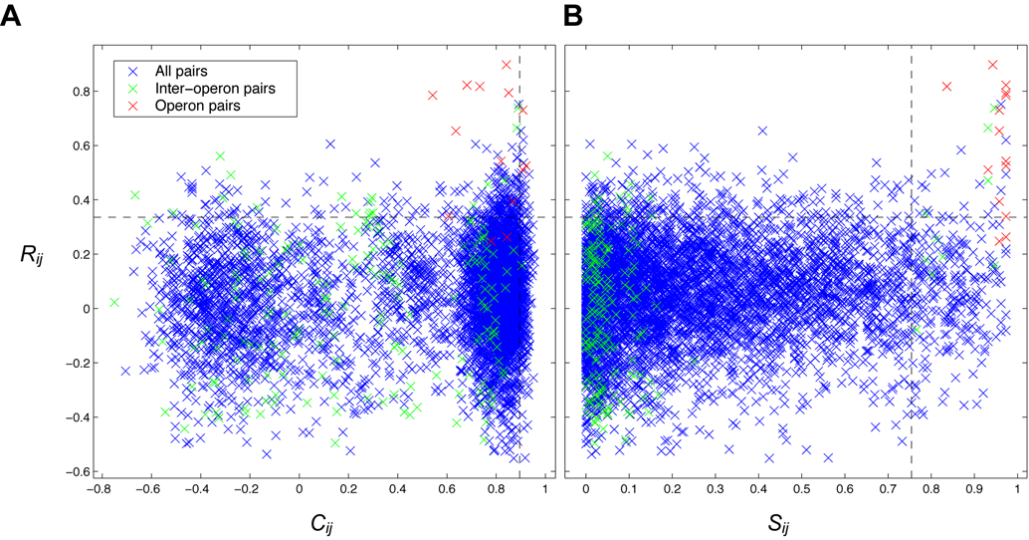
mRNA coexpression versus coevolution of *M. tuberculosis* PE/PPE gene pairs. Coevolution scores are shown on the *x* axis, mRNA coexpression scores are shown on the *y* axis. Red crosses represent pairwise scores for the 14 operon pairs; green crosses represent the 182 possible inter-operon pairs; blue crosses represent scores for the remaining 5394 genomewide PE/PPE pairs. Black dashed lines mark the upper 5% threshold for each score. (A) *R_ij_* vs. *C_ij_*, (B) *R_ij_* vs. *S_ij_*. In each of the panels, the region that is over the top 5% by both methods would be considered a combined prediction set. Notice that *S_ij_* offers superior operon pair recovery over *C_ij_*. Notice also that inter-operon pairs (green crosses) tend to have a low degree of coevolution (*C_ij_* and particularly *S_ij_*), implying negative selection against promiscuous interactions of operon paired PE and PPE proteins.

To confirm our intuition that predictions of PE/PPE coexpression and coevolution should overlap significantly, we analyzed the distribution of the two coevolution scores (*C_ij_* and *S_ij_*) combined with the coexpression score, *R_ij_*. The resulting distributions of the two score combinations are shown in Figure 4. In the figure, coevolution (*C_ij_* or *S_ij_*) is shown on the *x* axes; coexpression (*R_ij_*) is shown on the *y* axes. Red dots represent the 14 operon pairs; green dots represent the 182 inter-operon pairs (in which the PE and PPE are from different operon pairs); blue dots represent the other 5,394 genomewide PE/PPE pairs. Dashed lines are drawn to represent the upper 5% threshold for each score. Figure 4A illustrates our earlier assessment that *C_ij_* is not a useful prediction score due to the operon pairs not having a significantly different distribution from all PE/PPE pairs. Also notable in Figure 4A, only a minority of the operon pairs is found in the top 5% by both methods (3/14 operon pairs or 21%). Figure 4B shows better recovery of operon pairs in the top 5% by both methods (12/14 operon pairs or 86%). In light of these results, we concluded that the paralog matching score, *S_ij_*, is superior to *C_ij_* for predicting PE/PPE complexes. We therefore chose to combine *S_ij_* and *R_ij_* for subsequent predictions, and *C_ij_* was not used further in this study. The *S_ij_* and *R_ij_* scores for all PE/PPE pairs are provided in the Supporting Information (Dataset S3).

To assess the statistical significance of the overlap between predictions from coevolution and coexpression, we again employed a KS test. We asked whether the coexpression values of the top 5% coevolved PE/PPE genes (excluding the operon pairs) were higher than coexpression values of PE/PPE gene pairs overall. We found this to be the case (KS test, *P* = 0.02, α = 0.05, *k* = 0.09). From this we conclude that the PE and PPE proteins we predict to interact tend to be coexpressed, which we take as additional evidence for their possible interaction.

### Specificity of Operon Pair Interactions

To assess the specificity of interaction in PE/PPE operon pairs, we analyzed coevolution and coexpression of inter-operon pairs, the PE/PPE pairs in which both proteins are from different operon pairs. A KS test showed that inter-operon pairs (Figure 4, green crosses) had significantly lower score distributions than all other pairs by both coevolution scores, *C_ij_* and *S_ij_*, and the coexpression score, *R_ij_* (*P*≪0.0001 in all tests). We noticed a bimodal distribution of *C_ij_* (Figure 4A). Using a *k*-means algorithm we identified two clusters: a larger (4,208 protein pairs), positive-valued cluster with mean *C_ij_* = 0.78 and a smaller (1,382 pairs), negative-valued cluster with mean *C_ij_* = −0.21. The negative cluster contained no operon pairs, and was more than twice as likely to contain inter-operon pairs than the higher group (7% of the negative cluster; 3% of the positive one). We interpret these results as evidence of negative selection, both at the amino acid and gene expression levels, against cross-reactivity of operon paired PE and PPE proteins, and conclude that PE/PPE operon pairs, in general, interact specifically.

### Applying Our Method to Other Proteins

To demonstrate that our method is extensible to protein families other than PE and PPE we studied the ESAT-6/CFP-10 (Esx) family of proteins, which include some secreted antigens [39]. We chose the Esx family because they, like PE and PPE, tend to be found in operon pairs, some of which are known to code interacting proteins [40]–[42]. We applied our method to the 22 Esx proteins in *Mtb* H37Rv, in an identical manner to our analysis of the PE/PPE pairs, and found that known Esx interacting pairs and operon pairs, were given a high *S_ij_* (coevolution) by our method, and that many of these were supported by a high *R_ij_* (coexpression) (Figure S1). The *S_ij_* and *R_ij_* scores for the Esx analysis are given in the Supporting Information (Dataset 4). We conclude that our method has the potential to predict interactions in protein families beyond PE and PPE.

## Discussion

### The 35 Highest Confidence Predictions of PE/PPE Complexes

**Figure 5:**
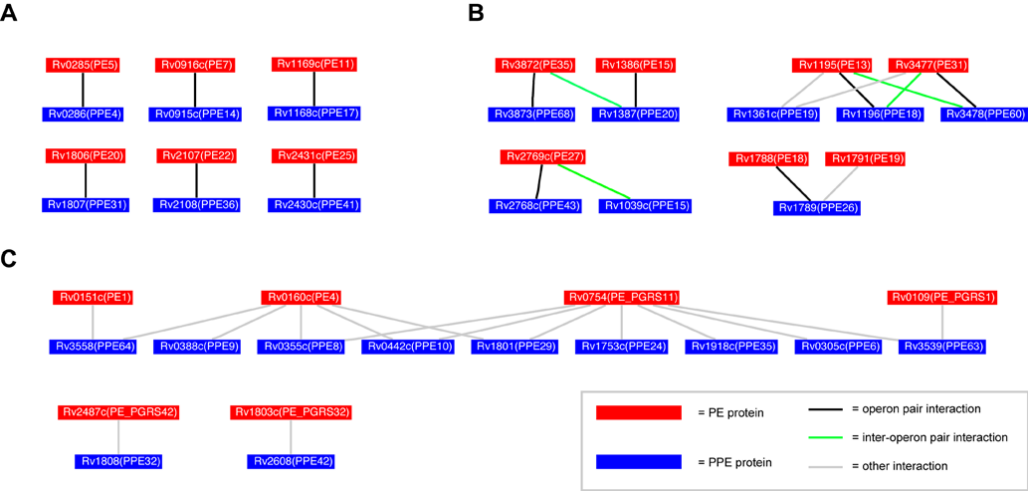
Network representation of predicted PE/PPE complexes. Proteins are nodes, interactions are edges. PE proteins are red; PPE proteins are blue. Proteins are labeled with their ORF numbers and gene names in parentheses. Operon pair interaction edges are black; inter-operon interactions are green; other interactions are gray. (A) PE/PPE operon pairs predicted to interact specifically. (B) Operon pairs with flexible interaction specificity, including some inter-operon pairings. (C) Interactions of PE_PGRS and other probable cell wall-associated PE proteins (Rv0151c and Rv0160c) with various PPE proteins.

To generate a high-confidence set of predicted PE/PPE complexes, we took the overlap of the top 5% by both coevolution (*S_ij_*) and coexpression (*R_ij_*), yielding the 35 pairs shown in Table 2. The same predicted interactions are shown in a network representation in Figure 5. Panel A shows that 6 of the 12 operon pairs in the top 35 predicted complexes are predicted to interact specifically. That is, the PEs in this group do not appear to interact with PPEs other than their operon partner, and vice versa. The specificity of interaction in operon pairs is also suggested by the tendency for inter-operon pairs to have low scores (Figure 4, green crosses). Figure 5B shows predicted cross-reaction of inter-operon pairs Rv3872/Rv1387, Rv1195/Rv3478, Rv3477/Rv1196, and Rv2769c/Rv1039c. Notice that the inter-operon interactions in Figure 5 are between pairs of proteins in the same colored regions in the phylogenetic trees in Figure 2, suggesting that pairs of paralogs with sufficiently similar sequences (nearby on a tree) could also cross-react. A high degree of mRNA coexpression (Table 2) provides additional evidence that there could be some cross reactivity between these PE/PPE operons. Cross-reactivity between genome-paired Esx proteins has been noted previously in *Mtb* [40], and it may be that the subunits of closely related PE/PPE complexes can similarly cross-react to confer functional flexibility as with the Esx family. However, our finding of negative coevolution and coexpression of the majority of the 196 possible inter-operon pairs (Figure 4) suggest that the four interactions we predict are exceptions rather than the rule.

**Table 2:** The 35 predicted PE/PPE complexes which are in the top 5% by both coevolution and coexpression methods.

PE ORF	PE gene	PPE ORF	PPE gene	Genomic distance (bp)	*S_ij_*	*R_ij_*	Operon pair
Rv3872	PE35	Rv3873	PPE68	31	0.97	0.78	o
Rv2431c	PE25	Rv2430c	PPE41	47	0.97	0.54	o
Rv2107	PE22	Rv2108	PPE36	56	0.97	0.34	o
Rv1806	PE20	Rv1807	PPE31	27	0.97	0.79	o
Rv1195	PE13	Rv1361c	PPE19	193141	0.97	0.62	
Rv1169c	PE11	Rv1168c	PPE17	18	0.97	0.82	o
Rv0916c	PE7	Rv0915c	PPE14	15	0.97	0.52	o
Rv0754	PE_PGRS11	Rv0355c	PPE8	411480	0.97	0.49	
Rv3477	PE31	Rv1361c	PPE19	2048983	0.96	0.75	
Rv2769c	PE27	Rv2768c	PPE43	80	0.96	0.39	o
Rv1386	PE15	Rv1387	PPE20	3	0.96	0.65	o
Rv1195	PE13	Rv1196	PPE18	47	0.96	0.73	o
Rv0160c	PE4	Rv0442c	PPE10	340312	0.96	0.43	
Rv3477	PE31	Rv1196	PPE18	1855889	0.95	0.74	x
Rv0754	PE_PGRS11	Rv1753c	PPE24	1133701	0.95	0.38	
Rv3477	PE31	Rv3478	PPE60	37	0.94	0.90	o
Rv2769c	PE27	Rv1039c	PPE15	1915686	0.93	0.47	x
Rv1788	PE18	Rv1789	PPE26	14	0.93	0.51	o
Rv1195	PE13	Rv3478	PPE60	1854325	0.93	0.66	x
Rv0754	PE_PGRS11	Rv1918c	PPE35	1319736	0.93	0.35	
Rv0754	PE_PGRS11	Rv1801	PPE29	1194088	0.91	0.46	
Rv1791	PE19	Rv1789	PPE26	1933	0.88	0.38	
Rv0160c	PE4	Rv1801	PPE29	1851562	0.87	0.58	
Rv2487c	PE_PGRS42	Rv1808	PPE32	744151	0.84	0.34	
Rv0285	PE5	Rv0286	PPE4	3	0.84	0.82	o
Rv0754	PE_PGRS11	Rv0305c	PPE6	470448	0.83	0.50	
Rv0160c	PE4	Rv0388c	PPE9	277020	0.83	0.34	
Rv0160c	PE4	Rv0355c	PPE8	234338	0.80	0.50	
Rv3872	PE35	Rv1387	PPE20	1621654	0.79	0.35	x
Rv1803c	PE_PGRS32	Rv2608	PPE42	888204	0.79	0.36	
Rv0754	PE_PGRS11	Rv3539	PPE63	1277590	0.79	0.45	
Rv0109	PE_PGRS1	Rv3539	PPE63	562813	0.79	0.35	
Rv0151c	PE1	Rv3558	PPE64	588834	0.78	0.45	
Rv0754	PE_PGRS11	Rv0442c	PPE10	313945	0.77	0.35	
Rv0160c	PE4	Rv3558	PPE64	600222	0.76	0.40	

In the operon pair column, o = operon pair and x = inter-operon pair. Notice that 12 of the 14 operon pairs are predicted in this set. Notice also the numerous PE and PPE genes which are not in the operon pairs, four inter-operon pairs and several members of the PE_PGRS subfamily.

Figure 5C shows possible cell surface-associated PE/PPE complexes. Four of the 6 PE proteins are PE_PGRS proteins (Rv0109, Rv0754, Rv1803c, and Rv2487c,), which are thought to be variable surface antigens displayed on the exterior of *Mtb* cells [5]. The other two PE proteins (Rv0151c and Rv0160c) were predicted in a previous study to contain membrane beta barrels [43], and thus are also likely localized to the cell surface. The PE and PPE proteins here appear to have multiple interaction specificities, particularly PE proteins Rv0160c and Rv0754, each of which is linked to several PPEs. Rv0160c and Rv0754 also have overlapping patterns of interaction as they share three predicted PPE partners: Rv0355c, Rv0442c, and Rv1801. However, in contrast to the example of Rv1195 and Rv3477 (Figure 5B), Rv0160c and Rv0754 do not have remarkably high sequence similarity (58%; both proteins are more similar to many other PEs) or close distance in the PE phylogenetic distance matrix. We would therefore conclude that the patterns of cross-reactivity shown in Figure 5C cannot be explained simply by sequence similarity. Future structural studies could reveal the detailed residue interactions responsible for complex formation among these proteins. Because of the possible cell surface localization of the proteins in Figure 5C, it may be that they are part of multiprotein cell-surface complexes involving varying combinations of PPE proteins interacting with surface-localized PE proteins. We truncated all proteins to include only the PE or PPE domains; therefore our method predicts that PE_PGRS and membrane-associated PE proteins interact with PPEs through their PE domains. All of these interpretations await experimental confirmation.

Two of the 14 *Mtb* H37Rv operon pairs (Rv1040c/Rv1039c and Rv3622c/Rv3621c) were not among the 35 putatively interacting PE/PPE pairs identified by our procedure. Both of these operon pairs exceeded our coevolution (*S_ij_*) threshold, but were slightly below our coexpression (*R_ij_*) threshold of 0.34 (*R_ij_* = 0.26 and 0.25, respectively; see also Figure 4). Employing a lower *R_ij_* threshold, for example the 85^th^ percentile (*R_ij_* = 0.24), would result in the predicted interaction of all 14 operon pairs.

### Including Non-Interface Residues in Complex Predictions

To predict new PE/PPE interactions we analyzed sequences homologous to the interacting domains in the known PE/PPE complex [13], without explicitly limiting our analysis to defined interface residues between the subunits as in [44]. We reasoned that, because both PE and PPE are helical, that in different paralogous complexes the registry of the helices could change, bringing different residues into the interface. This may be especially true for PE and PPE, which are highly elongated in shape (the characterized PE/PPE complex is about 108 Å long by 26 Å wide [13]), and, as a result, most residues in either subunit are near (within 10 Å) a residue in the other subunit. Computational and experimental studies [29],[45] have found evidence for energetic coupling of distant residues (>10 Å) in a number of protein families. While these studies focused on protein monomers, it is possible that the finding of long-range residue-residue interactions could also apply to complexes. With this in mind, we were cautious about excluding regions homologous to either subunit in which a mutation might not be ‘felt’ in the other subunit.

To test whether excluding residues distant from the complex interface would influence our results, we inspected the PE/PPE structure [13] and found a contiguous region spanning residues 101–148 of the PPE with C-α distances greater than 10 Å from the nearest residue of the PE. We constructed a modified PPE alignment omitting these residues (and homologous regions of aligned PPE proteins) and repeated our analysis, and found no significant change in the distance matrix made from the unmodified PPE alignment (correlation of PE and modified PPE distance matrices = 0.84; no improved correlations in 10^6^ matrix shufflings). We conclude that including some non-interface residues, at least in the case of PE/PPE, did not significantly bias our results.

### Predicting Complexes from One or Many Genomes

The large expansion of the PE and PPE genes in *Mtb* [12] allowed us to obtain results using the genome sequence of *Mtb* strain H37Rv alone, without bringing in data from other genomes. Our method could therefore in principle be applied to large families of interacting paralogs in microbial genomes without necessarily having any closely related genome sequences from other microbes.

To explore whether adding genome data from other mycobacteria would improve our results, we searched an additional 14 mycobacterial genomes for PE/PPE operon pairs. Orthologs to H37Rv PE/PPE operon pairs are summarized in Figure S2. Ninety additional operon pairs were found, adding to the 14 of *Mtb* H37Rv to give 104 operon pairs in total. However, only 24 of these operon pairs had PE or PPE domains with amino acid sequences different (usually just by an amino acid or two) from the 14 H37Rv operon pairs. The 38-protein reference matrices derived from multiple genomes showed a reasonable increase in correlation over the 14-protein reference matrices from H37Rv (0.91 up from 0.84; no higher correlations yielded by shuffling the matrices' gene order 10^6^ times). No clear improvement in the results was evident from using the multi-genome set of operon pairs (data not shown), perhaps because each of the added 24 sequences was highly similar to one already in the 14 H37Rv operon pairs. However, it is likely that as still more mycobacterial genomes are sequenced, new sequence variants of PE and PPE domains will be discovered, and this may improve our results.

In the five *Mtb* strains analyzed, 65/70 or 93% of the operon pairs were conserved (Figure S2). Five operon pairs were missing either the PE or PPE protein. In particular, the *Mtb* C strain has single-gene deletions in three operon pairs (Rv1040c/Rv1039c, Rv1806/Rv1807, and Rv2107/2108. It is possible that in *Mtb* strains with ‘broken’ operon pairs, another interacting partner is able to interact with the orphaned gene, possibly restoring the PE/PPE complex's function, or introducing new complexes that help these strains survive in their environmental niches.

It is possible that a single *Mtb* strain (in this case H37Rv), in which the PE and PPE families are more expanded relative to other mycobacteria [12], provides a broad sampling of the tolerated residue variations in these families, as proteins with many paralogs are thought to be under negative selective pressure on their interactions with paralogs other than their cognate partner [46]. Thus, the more specifically interacting PE/PPE protein pairs there are in a genome, the more residue variation we might see, due to positive selective pressure on interaction specificity. The extent of interaction promiscuity between the PE and PPE families is unknown, but our observations are consistent with negative selection on promiscuous interaction. This suggests that there may be some advantage to *Mtb* in maintaining interaction specificity of PE and PPE proteins, at least in those that are operon-paired (and which we also predicted to interact with some degree of specificity, Figure 5A). We conclude that our analysis was not significantly affected by the inclusion of only a single genome, and that this could be a useful approach for mining the interactions of newly sequenced genomes for which there are initially no (or just a few) related genomes to compare to.

### Differences of Our Method with Related Methods

Related prediction methods [20],[21],[24] that compared phylogenetic distance matrices relied on a training set of experimentally determined protein-protein interactions to build the reference matrices, or large sets of orthologs of a few known interacting proteins [25]. At the time of this study, there is only a single experimentally characterized PE/PPE interaction [13]. We resolved this impasse by employing the operon method [15]–[18] to define a high-confidence set of predicted interactions (including the known complex) to build phylogenetic distance matrices capable of capturing some of the residue covariation patterns in PE/PPE complexes. The validity of our results depends on future verification of this assumption. Even so, the high degree of coevolution and coexpression seen in operon-paired PE/PPE genes, combined with definitive experimental characterization of at least one PE/PPE complex [13], implies that our assumption that operon-paired PE/PPE genes code complexes is fair.

### Testing Predictions

We envision testing the PE/PPE complexes predicted by our approach with a scalable high-throughput strategy. Rapid cloning methods such as ligation-independent cloning (LIC) [47]–[49] could be employed to rapidly build up PE/PPE co-expression plasmids like that described for the Rv2431c/Rv2430c complex [13]. These strategies allow the experimentor to avoid time-consuming restriction and ligation steps during cloning. Using LIC, putative PE/PPE complexes could rapidly be screened to assess whether further study, including structural characterization, would be worthwhile. Using our set of predicted interactions to prioritize experiments would likely reduce the required number of assays to successfully characterize complexes. As all PE/PPE pairs in our set of operon pairs were found in the top 5% of predictions it is possible that the success rate of such prioritized assays, relative to all-vs-all screening, could be increased by an order of magnitude or more.

### Conclusions

A method for predicting the interactions of the PE and PPE families of proteins in *M. tuberculosis*, beyond those simply linked by the operon method, is proposed. The method combines known interacting domain structure, genomic operon organization, and protein coevolution, and predicts that 35 pairs of PE and PPE proteins interact. Our method can be applied to a single genome if sufficient numbers of paralogs are present, or could be used in a multi-genome framework. A subset of the predictions from our coevolution-based method is confirmed by high mRNA coexpression, suggesting their biological relevance, and likely weeding out false positives. Our results may be a useful starting point for experimentally probing the interactions of PE, PPE, and other microbial protein families.

## Materials and Methods

### Identifying Same-Operon PE/PPE Gene Pairs

Annotations for the *Mtb* H37Rv genome were downloaded from the NCBI FTP site (ftp://ftp.ncbi.nih.gov). PE and PPE genes were identified from these annotations. The gene coordinates and orientation information provided in the annotations were used to compile a list of adjacent PE/PPE pairs in the same orientation, with the PE protein located 5′ (upstream) to the PPE protein, and with no more than 100 base pairs intergenic separation. Increasing the intergenic distance cutoff to 500 base pairs did not result in any additional PE/PPE pairs.

### Analysis of *E. coli* Complexes and Operons

280 multiprotein complexes involving 692 proteins, and 2,909 unique operons involving 4,510 genes, were extracted from the EcoCyc database [30]. A total of 1918 pairwise protein interactions were found in the complexes. Of these pairs, 942 or 49% of the pairs were also found together in an operon. To assess the significance of the overlap, the identities of the proteins in the complexes were randomized (each protein was replaced with a unique, random *E. coli* protein), and the co-occurrence in operons was reassessed. One thousand shuffling trials were performed and the overlap of 49% was not met or exceeded in any of the trials. The maximum overlap achieved in any trial was 2%.

### Structure-Based Multiple Alignments of PE and PPE Families

For each of the PE and PPE families, family member sequences were selected from the SwissProt database [50] by two criteria: 1. the sequence was annotated as belonging to either the PE or PPE protein families in Pfam, or 2. the protein was otherwise annotated as belonging to one of these families. Multiple alignment of the protein sequences was performed using the ClustalW program using default parameters, and a secondary structure profile generated by the DSSP program [32] and the known structure [13]. Alignments were visually inspected and hand-edited to omit sequences with obvious low homology. Rv3893c (PE36), though classified by SwissProt as a PE protein, appeared to be an outlier and was therefore removed from the multiple alignment. Rv3892c (PPE69), its genome-paired neighbor, was kept in the PPE multiple alignment. Because of the omission of Rv3893c, the PE/PPE pair of Rv3893c/Rv3892c was not included in the genome-paired reference set. Rv3892c was included in subsequent predictions.

The resulting structure-based alignments had 87 proteins in the PE alignment, and 65 proteins in the PPE alignment. Multiple alignments were truncated to include only those positions homologous to proteins in the known PE/PPE complex of Rv2431c/Rv2430c. Rv2431c and Rv2430c are among the shortest members of the PE and PPE families, respectively, and inspection of the complex structure suggests that these sequences may represent the minimal interface regions necessary to form a complex. Many PE and PPE proteins have other domains, low-complexity regions, and transmembrane domains C-terminal to their PE or PPE domain. These regions are unlikely to participate directly in the PE/PPE interaction; this was our reasoning for removing these regions from the alignment.

### Making Phylogenetic Distance Matrices for the PE and PPE Families

From the full-family multiple alignments, Phylip distance matrices were generated in the ClustalW program [31]. This resulted in an 87×87 matrix for the PE family and a 65×65 matrix for the PPE family. These matrices would be used subsequently in predictions to give us the distance between any pair of PE sequences and any pair of PPE sequences.

Next, phylogenetic distance matrices were made for just the 14 pairs of operon-paired PE and PPE proteins. This was done by extracting the 14 operon-paired sequences from each of the full-family multiple alignments. The resulting subalignments were used to generate a 14×14 PE distance matrix and a 14×14 PPE matrix. Importantly, the two 14×14 matrices were ordered so that the *i*th protein in the PE matrix was the operon partner of the *i*th protein in the PPE matrix. We would later use these matrices as ‘reference’ matrices for prediction of non-operon-paired PE/PPE complexes.

### Phylogenetic Trees of PE and PPE Families

Phylogenetic trees were generated from the PE and PPE 14-sequence subalignments using the ClustalW program [31]. The correction for multiple substitutions was not used to generate the trees. Bootstrapping of the trees was done within the ClustalW program.

### Inferring PE/PPE Complexes from Coevolution

Let ***X*** be the 14×14 distance matrix of PE proteins, and ***Y*** be the 14×14 distance matrix of PPE proteins. *X_ij_* is the percent divergence of PE*_i_* and PE*_j_*; likewise *Y_ij_* is the percent divergence of PPE*_i_* and PPE*_j_*. *X_i_* is the vector of length 14 for the distances of PE*_i_* from all PE*_j_* (including for *i* = *j*, in which case *Y_ij_* = 0.0); likewise *Y_i_*.

To determine the correlation between the ordered 14×14 distance matrices, ***X*** and ***Y***, the Pearson correlation is taken. To avoid counting protein distances twice, only the unique elements in the matrices are taken (that is, we count *i*,*j* pairs but not *j*,*i*).
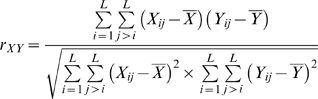

*L* = 14, the number of operons with paired PE and PPE genes. *r_XY_* is a measure of the coevolution of the operon-paired subsets of the PE and PPE protein families.

Next we derive *C_ij_*, a measure of the coevolution of PE*_i_* with PPE*_j_*, for all PE and PPE proteins in the *Mtb* genome, including but not limited to proteins in the operon-paired set. For PE*_i_*, we generate *A_i_*, a distance vector of length 14, containing the distances from PE*_i_* to each of the 14 PE*_k_* in the 14×14 reference matrix. *B_j_* is equivalently generated for PPE*_j_*. To get *C_ij_*, a measure of the coevolution of PE*_i_* with PPE*_j_*, the Pearson correlation of *A_i_* and *B_j_* is calculated.
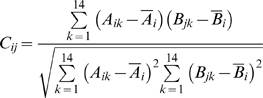
Note that here we are taking the correlation of two vectors with length 14.

To generate the paralog matching score, *S_ij_*, a reciprocal ranking procedure was used. For each PE, a ranked list of the most coevolved PPEs was produced. The same was done for PPEs. Then, for each PE*_i_*, we recorded the position of each PPE*_j_* protein in the PE's list of PPEs ranked by *C_ij_* to give *r_i_*_→*j*_, the number of PPEs ranking below PPE*_j_*. The reciprocal procedure was performed to yield *r_j_*_→*i*_.
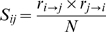

*N* is the total number of PE/PPE pairs. Using the above formula, a high score is assigned only if both pairs were high on each other's list of most coevolved potential interacting partners.

### Prediction Evaluation

Positive examples of PE/PPE complexes were defined as all of the 14 operon-paired proteins. A dataset of negative PE/PPE interactions are not currently available, so we made the assumption that operon-paired PE/PPE interactions were specific, and therefore a PE in one operon would not interact with a PPE in a different operon. 182 inter-operon PE/PPE pairs resulted, and were used as a negative set.

Next, for a range of prediction thresholds from 0.0 to 1.0, true positive (*TP*), true negative (*TN*), false positive (*FP*), and false negative (*FN*) rates were determined. The sensitivity, or true positive rate (*TPR*), was calculated as
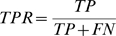
1-specificity, or the false positive rate (*FPR*), was calculated as
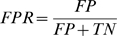



In a receiver operator characteristic curve (not shown), the prediction threshold corresponding to the upper left-most portion of the curve represents the optimum compromise between TPR and FPR. For prediction with *S_ij_*, this threshold was roughly 0.75. Taking all PE/PPE pairs with *S_ij_*≥0.75 gives roughly 5% of the total 5,590 possible PE/PPE pairs, in which all 14 of the operon pairs were included.

### Analysis of mRNA Coexpression of PE and PPE Genes

Nine *Mtb* gene expression datasets (Table S1) in the Gene Expression Omnibus (GEO) [38] were downloaded. All available *Mtb* datasets in GEO were used excluding for consistency those that studied deletion mutants or attenuated strains. Also for consistency, only datasets that reported gene expression changes as a ratio of a sample and a reference were used. Gene expression data from the nine studies were represented as a matrix where the rows were genes and the columns were experiments. To construct an expression vector for a gene, the data from each of the nine studies were concatenated. Combined expression vectors were made up from the field labeled ‘VALUE’ in the data files. In all data sets, this value represents the measured expression level of a gene under experimental conditions versus that gene in a reference sample. Various normalization schemes were applied by the authors of the individual datasets to correct for scale differences due to differing intensities among genes in response to different experiments. Because of the difficulty in combining these schemes to make a self-consistent combined dataset, we chose not to further normalize the expression data.

Correlation coefficients of gene expression vectors were calculated for all possible pairs of genes. To obtain a correlation coefficient for genes *i* and *j* over *N* experiments, the Pearson correlation coefficient, *R_ij_*, was used.
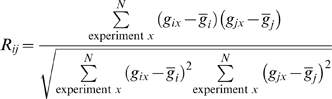
where *g_ix_* and *g_jx_* are the expression values reported in the GEO data file for genes *i* and *j*, respectively, in experiment *x*. For each pair of genes analyzed, combined expression vectors were truncated by deleting experiments in which either or both genes had missing values. Thus *N* varied for each pair of genes assessed. In all, 734 experiments were used for the inference of pairwise coexpression relationships between pairs of PE and PPE genes.

### Statistical Significance Tests

The Kolmogorov–Smirnoff (KS) test asks whether two collections of random samples come from the same distribution. We want to know if the coexpression scores for a group of PE/PPE gene pairs predicted to code interacting proteins has a different distribution (with a higher mean) than PE/PPE gene pairs overall. Because we expect the linked proteins to have a higher-valued mean than the unlinked proteins, we used the one-tailed version of the KS test. An α significance criteria of 0.05 was used for hypothesis acceptance/rejection.

### Structural Analysis of PE/PPE Complex

The PE/PPE complex described in [13] was analyzed using the RasMol program [51]. The structure was visually inspected to identify a contiguous region spanning residues 101–148 of the PPE with C-α distances greater than 10 Å from the nearest residue of the PE. The PPE multiple alignment was modified by deleting all columns that aligned to this contiguous region.

### Interactions of ESAT-6/CFP-10 (Esx) Paralogs

The 22 Esx family genes were identified in *Mtb* H37Rv from NCBI annotations. The genes were divided into two groups: ESAT-6 paralogs (12 proteins) and CFP-10 paralogs (10 proteins). We based this categorization on the observation that 20 of the 22 the Esx genes are organized into 10 adjacent (operon) pairs on the genome, with a gene similar to CFP-10 lying upstream from a gene similar to ESAT-6. We therefore categorized upstream genes as CFP-10 paralogs and downstream genes as ESAT-6 paralogs. The two annotated Esx genes not in operon pairs, Rv1793 and Rv3017c, were both judged to be ESAT-6 paralogs from visual inspection of a multiple alignment. The 10 Esx operon pairs were used to build reference matrices as with PE and PPE. Coevolution (*S_ij_*) and coexpression (*R_ij_*) scores were derived using the same procedure as for PE and PPE.

### Detection of PE/PPE Operon Pair Orthologs in Multiple Genomes

For each PE or PPE gene in an operon pair, orthologs were manually extracted from the TB Database (http://www.tbdb.org). The results are summarized in a tab-delimited file in the Supporting Information (Dataset 5).

### Proteins Analyzed

The ORF identifiers, gene names, and SwissProt accession codes of the PE and PPE proteins analyzed in this study are listed in Text S1.

## Supporting Information

Dataset S1 Structure-based alignment of PE proteins.
(0.04 MB DOC)

Dataset S2 Structure-based alignment of PPE proteins.
(0.05 MB DOC)

Dataset S3 Paralog matching scores *S_ij_*, coexpression scores *R_ij_*, and operon pair information for all PE/PPE pairs.
(0.32 MB TXT)

Dataset S4 Paralog matching scores *S_ij_*, coexpression scores *R_ij_*, and operon/interaction information for Esx proteins.
(0.00 MB TXT)

Dataset S5 For each PE or PPE gene in an operon pair, orthologs in mycobacterial genomes were manually extracted from the TB Database (http://www.tbdb.org). The results are summarized in this tab-delimited file.
(0.00 MB TXT)

Figure S1 Predicted interactions of ESAT-6/CFP-10 (Esx) family proteins. Interactions are scored by *S_ij_* and *R_ij_*. Esx pairs not in operons are shown as blue crosses; operon pairs are green; operon pairs found in experiments to interact are red; non-operon pairs found to interact are magenta. Notice that all known interactions (red and magenta) and nearly all operon pairs (green) tend to be highly scored by our method (tending towards the upper right of the plot), suggesting our method's applicability to other protein families.
(11.34 MB TIF)

Figure S2 Comparative genomic analysis of PE/PPE operon pairs. The ORF identifiers of PE/PPE operon pairs from H37Rv are shown on the left on alternating gray and white backgrounds. Genomes containing orthologous operon pairs are shown at the top, with *Mtb* genomes shaded in cyan. Red rectangles show the presence of PEs; blue show PPEs. White spaces indicate that no ortholog was found in that genome. Notice that the PE/PPE pairs appear well-conserved in *Mtb* and *M. bovis* strains, and that a few operon pairs are disrupted by the loss of a gene in the *Mtb* CDC1551, C, and F11 strains.
(16.58 MB TIF)

Table S1 Gene expression datasets used.
(0.04 MB DOC)

Text S1 Accession numbers. ORF identifiers, gene names, and SwissProt accession codes for proteins analyzed in this study.
(0.14 MB DOC)
