# Supplementary material for: Identifying Cognate Binding Pairs among a Large Set of Paralogs: The Case of PE/PPE Proteins of Mycobacterium tuberculosis
Source: PLoS Comput Biol. 2008 Sep 12;4(9):e1000174. doi: 10.1371/journal.pcbi.1000174 (PMC2519833; doi:10.1371/journal.pcbi.1000174)
Supplement: Dataset S1 — Structure-based alignment of PE proteins. (0.04 MB DOC) [file pcbi.1000174.s001.doc]

CLUSTAL W (1.83) multiple sequence alignment

!SS_Rv2431c .aaaAAAAAAAAAAAAAAAAAAAAAAaaa.---........aaaAAAAAAAAAAAAAAAA

Rv2431c PEALTVAATEVRRIRDRAIQSDAQVAPMTT---AVRPPAADLVSEKAATFLVEYARKYRQ

Rv1788 PEALAAAAGSLQGIGSALNAQNAAAATPTT---GVVPAAADEVSALTAAQFAAHAQIYQA

Rv1791 PEALAAAAANLQGIGTTMNAQNAAAAAPTT---GVVPAAADEVSALTAAQFAAHAQMYQT

Rv1195 PEMLAAAADTLQSIGATTVASNAAAAAPTT---GVVPPAADEVSALTAAHFAAHAAMYQS

Rv3622c PEMLAATAGELQSINAVARAGNAAVAGPTT---GVVPAAADLVSLLTASQFAAHAQLYQA

Rv3477 PEMLAAAAGELRSLGATLKASNAAAAVPTT---GVVPPAADEVSLLLATQFRTHAATYQT

Rv2769c PEELAAAAGKLETIGSAMVAQNAAAAAPTTT--GVIPAAADEISVLQAPLFTAYGTLYQQ

Rv1040c PEELTAAAAQLGTIGAAMAAQNAAAAAPTT---AIAPAALDEVSALQAALFTAYGTFYQQ

Rv0742 PEAIAAAATDLASIGSTIGAANAAAAANTT---AVLAAGADQVSVAIAAAFGAHGQAYQA

Rv1818c PEALAAVATDLAGIGSTIGTANAAAAVPTT---TVLAAAADEVSAAMAALFSGHAQAYQA

Rv0278c PEVIAAAATDLASLGSSISAANAAAAANTT---ALMAAGADEVSTAIAALFGAHGQAYQA

Rv1759c PETIAAAATDLADLGSTIAGANAAAAANTT---SLLAAGADEISAAIAALFGAHGRAYQA

Rv0747 PELVVAAAADLAGIGSAISSANAAAAVNTT---GLLTAGADEVSTAIAALFGAQGQAYQA

Rv0279c PEVIAAAATDLASLESSIAAANAAAAANTT---ALLAAGADEVSTAVAALFGAHGQAYQA

Rv1325c PETLVRAASDLANIGSTLGAANAAALGPTT---ELLAAGADEVSAAIASLFAAHGQAYQA

Rv1214c --MLASAATDLAGIGSALSAANAAAAAPTT---AMLAACADEVSAVVASLFARHAQAYQA

Rv2741 PEFLTAAAMDLASIGSTVSAASAAASAPTV---AILAAGADEVSIAVAALFGMHGQAYQA

Rv2634c PEALTMAASDLANIGSTINAANAAAALPTT---GVVAAAADEVSAAVAALFGSYAQSYQA

Rv1396c PEMLGAAATDLASIGSAISTANAAAAAATT---RVLAAGADEVSAAVAALFSGHAQTYQA

Rv0124 PEIVVAAATDLAGIGSAISAANAAAAAPTT---AVLAAGADEVSAAIAALFSGHAQAYQA

Rv3595c PEFLSAAATDLANLGSTISAANAAASIPTT---GVLAAGADDVSAAIAALFGAHAQAYQT

Rv2396 PEALAATATYLTGIGSAISAANAVAAAPTT---EILAAGTDEVSTAISALFGAHAQAYQA

Rv1468c TEFVSGAAGNLARLGSMISAANSAAAAQTT---AVAAAGADEVSAAVAALFGAHGQTYQV

Rv1091 PEALVAVASDLAGIGSALAEANAAALAPTT---ALLAAGADEVSAAIAALFGAHGQAYQT

Rv0832 PATLATAATEVARIGSALSLASAVAAAQTS---AVQAAAADEVSAAIAALFSAHGRDFQA

Rv2162c PEVMAAAATDLANIGSSISAASAAAAGPTM---GILAAGADEVSVAISALFGSHAQGYQT

Rv2490c PEMMATAAFDLARIGSQVSAASAVAAMPTT---EVVAAGADEVSAGIAALFSAHAQEYQA

Rv1087 PEVLAAAASDLAGIGSTLAQANAAALAPTT---AVLAAGADEVSAAIASLFGAHGQAYQA

Rv1068c PDMLSSAAGDLASIGSSINASTRAAAAATT---RLLPAAADEVSAHIAALFSGHGEGYQA

Rv1067c PSQLMAAAADVAGIGSAISAANAAALAPTS---VLAAAGADEVSAAVAALFSAHAGQYQQ

Rv1840c PEVVVAAASDLAGIGSAIGAANAAAAVPTM---GVLAAGADEVSAAVADLFGAHAQAYQA

Rv0578c PEMLTTAATDLAKIGSTITAANTAAAAVAK----VLPASADEVSVAVAALFGTHAQEYQT

Rv2615c PQLVSTAAADAARIGSAINTANTAAAATTQ----VLAAAQDEVSTAIAALFGSHGQHYQA

Rv1768 PELVAAAATDLANIGSSISAANAAAAAPTT---ALVAAGGDEVSAAIAALFGAHARAYQA

Rv0109 PATVAAAATHLAGIGSALSTANAAAAAPTT---ALSVAGADEVSVLIAALFEAYAQEYQA

Rv3590c PEALMSVASEVAGIGSALNAANAAAAAPTT---GVLAAAADEVSAAMAALFGAHAQEYQR

Rv2098c PEALLAAATDLAAIRSTIRAANAAAAVPTT---GALAPAADEVSAGIAALFGAQAQSYQA

Rv3508 PEFVTAAAGDLTNLGSSISAANASAASATT---QVLAAGADEVSARIAALFGGFGLEYQA

Rv3514 PEFVTAAAGDLTNLGSSISAANASAASATT---QVLAAGADEVSARIAALFGGFGLEYQA

Rv3388 PEMLAAAATDLAGIRSAISAATAAAAAPTI---QVAAAGADEVSLAISALFGQHAQAYQA

Rv3345c PELVAAAAADLTGIGQAISAANAAAAGPTT---QVLAAAGDEVSAAIAALFGTHAQEYQA

Rv1450c PETVAAAALDVARIGSSIGAANAAAAGSTT---SVLAAGADEVSAAIATLFGSHAREYQA

Rv1452c PETVAAAASDVARIGSSIGVANSAAAGSTT---SVLAAGADEVSAAIATLFGSHAREYQA

Rv1441c PGMLSAAAADVASIGAALSAANGAAAPTTA---GVLAAGADEVSAAIASLFSGYARDYQA

Rv0746 PEVLGSAATDLAALGSVLGAADAAAAATTT---GIVAAAQDEVSAAIAALFSAHGRAYQV

Rv3650 PEALDSAATDLVVLGSTLGAATAAAAAQTT---GIVAAAHDEVSAAIAALFSAHGQAYQA

Rv2591 PEMLATAAQNVANIGTSLSAANATAAASTT---SVLAAGADEVSQAIARLFSDYATHYQS

Rv3511 PEVVSAAAGDLANVGSTISAANKAAAAATT---QVLAAGADEVSARIAALFGMYGLEYQA

Rv0532 PELVAAAAADLAGIGSAIGAANAAAGAPTM---ALLAAGADEVSAAVAAVFSSYAQQYQA

Rv1243c QDVLVAAAADLEGIGSALAAANRAAEAPTT---GLLAAGADEVSAAIASLFSGNAQAYQA

Rv0977 PPVLASAASDLGGIASMISEANAMAAVRTT---ALAPAAADEVSAAIAALFSSYARDYQT

Rv0872c PEMVAAAANNLAQIGSTLSAANAAALAPTT---GVLAAGADEVSAAVASLFSGHAQAYQT

Rv2853 PDLMTAAATNLAEIGSAISTANGAAALPTV---EVVAAAADEVSTQIAALFGAHARSYQT

Rv0834c PDLVAMATEDLAGIGASLTAANAAAAVPTS---GLLAAAGDEVSAAIAALFSSHGQQYQA

Rv2487c PQLLATAALDLASIGSQVSAANAAAAMPTT---EVVAAAADEVSAAIAGLFGAHARQYQA

Rv3507 PETVAAVATDLKRIGASLAHENASAAASTT---AVVSAAADEVSTAVAALFSQHAQGYQA

Rv3367 PEALAAAASDVANIGSALSAANAAAAAGTT---GLLAAGADEVSAALASLFSGHAVSYQQ

Rv0297 PEMIAAAAGELASIRSAINAANAAAAAQTT---GVMSAAADEVSTAVAALFSSHAQAYQA

Rv1803c PAFVDAAAKDLATIGSAISRANAEALVPIT---ALLPAGADDVSAAIAALFATHGQAYQE

Rv1806 PDALAIAAGQLRHVGSVIAARNAVAAPATA---ELAPAAADEVSALTATQFNFHAAMYQA

Rv1651c PDLVTAAAANLAGIRSALSEAAAAASTPTT---ALASAGADEVSAAVSRLFGAYGQQFQA

Rv0978c PQLVSTAAADAARIGSAINTANTAAAATTQ----VLAAAQDEVSTAIAALFGSHGQHYQA

Rv0980c PQLVSTAAADAARIGSAINTANTAAAATTQ----VLAAAHDEVSTAIAALFGSHGQHYQA

Rv0916c PVVLAAATGDLPTIGTAVSARNTAVCAPTT---GVLPPAANDVSVLTAARFTAHTKHYRV

Rv2340c PNVLAASAGELAAIGSTMRAANAAAAAPTA---GVLAAGGDDVSAGIAALFGARAQAYQA

Rv1983 PEFLTSAAADVENIGSTLRAANAAAAASTT---ALAAAGADEVSAAVAALFARFGQEYQA

Rv0754 RDALAAAAADLAQIGSAVNAGNLAAANPTT---AVAAAAADEVSAALAALFGAHAREYQA

Rv2107 PFGMLAAAATLESLGSHMAVSNAAVASVTT---KVPPPAADYVSKKLSLFFSSHGQQYQV

Rv2519 PDWLASAAAEVQSIGSALSAANAAAAAPTT---LLVAAAEDEVSAAAAALFANYGREYQT

Rv1088 PAALTAAATDIDGIGSAVSVANAAAVAATT---GVLAAGGDEVLAAIARLFNANAEEYHA

Rv3652 PEALVAAATDLATLGSTIGAANAAAAGSTT---ALLTAGADEVSAAIAAYSECTARPIRH

Rv3022.1 PEGLAAASAAVEALTARLAAAHAGAAPAIT---AVVAPAADPVSLQSAVGFSALGSEHAA

Rv0285 PEGLAAASAAVEALTARLAAAHASAAPVIT---AVVPPAADPVSLQTAAGFSAQGVEHAV

Rv1386 PESLAGASAAIEAVTARLAAAHAAAAPFIA---AVIPPGSDSVSVCNAVEFSVHGSQHVA

Rv1172c PEALAAAAADMAGIGSTLNAANVVAAVPTT---GVLAAAADEVSTQVAALLSAHAQGYQQ

Rv2328 PEQVESAAQDLAGIRSALSASYAAAAGPTT---AVVSAAEDEVSTAIASIFGAYGRQCQV

Rv1169c PDSIGETAANLHEIGVTMSAHDDGVTPLIT---NVESPAHDLVSIVTSMLFSMHGELYKA

Rv1646 PDMVTAAAGNLESVGSALNEAAAAAAPATV---GLAAPAADRVSAVVAAMLGAYARDFQG

Rv2408 PDILCSRGPEAMRAKAADLDLAAAAKTVGV------QPAADQVAAAIAAILLSHAQIYQD

Rv0335c PGPLCATTRPRPPQGNQPPASRISNFPPKRHKTRVLAAAEDEVSAAVAALISAHGRRHHS

Rv3097c PEVMSAAATDVASIGSVVATASQGVAGATT---TVLAAAEDEVSAAIAALFSGHGQDYQA

Rv1430 PEMVAATASDLASLGAALSEATAAAAIPTT---QVLAAAADEVSAAIAELFGAHGQEFQA

Rv0151c PSVLAAAATDLAGIGSAINQATAAVAAPTT---GLAAAAADEVSTALATLFGAYGQQFQA

Rv0160c PDMLATAAAHVDEIASTLRAANAAAAGPTCN---LLAAAGDEVSAATAALFSAYGREYQA

Rv0159c PEMLATTAADVDGIGSAIRAASASAAGPTT---GLLAAAADEVSSAAAALFSEYARECQE

Rv3812 PEAVAAAAGDLAAIGSTLREATAAAAGPTT---GLAAAAADDVSIAVSQLFGRYGQEFQT

Rv3872 PIAADIGTQVSDNALHGVTAGSTALTSVTG----LVPAGADEVSAQAATAFTSEGIQLLA

. : : :

!SS_Rv2431c AAAaaa-aaaAAAAAaaa...

Rv2431c TIAAAA-VVLEEFAHALTTGA

Rv1788 VSAQAA-AIHEMFVNTLQMSS

Rv1791 VSAQAA-AIHEMFVNTLVASS

Rv1195 VSARAA-AIHDQFVATLASSA

Rv3622c ISAEAM-AVQEQLATTLGISA

Rv3477 ASAKAA-VIHEQFVTTLATSA

Rv2769c VSAEAA-AVYDLFVKTLGVSA

Rv1040c VSAEAQ-AMHDMFVNTLGISA

Rv0742 LSAQAA-TFHIQFVQALTAGA

Rv1818c LSAQAA-LFHEQFVRALTAGA

Rv0278c LSAQAQ-AFHAQFVQALTSGG

Rv1759c ASAEAA-AFHGRFVQALTTGG

Rv0747 ASAQAA-AFYAQFVQALSAGG

Rv0279c LSAQAQ-AFHAQFVQALTSGG

Rv1325c VSAQMS-AFHAQFVQTFTAGA

Rv1214c LSLQAT-AFHQQFVQALTGAG

Rv2741 LSVQAS-AFHQQFVQALTAGA

Rv2634c FGAQLS-AFHAQFVQSLTNGA

Rv1396c LRTQAA-AFHQQIVQTLTSTA

Rv0124 LSAQAA-AFHQQFVQTLAGGA

Rv3595c ISAQAA-TFHAQFVQTLSAGA

Rv2396 LSAHVA-AFHDQFVHTLTAGA

Rv1468c LSAQAA-AFHSQFVQALSGGA

Rv1091 VSAQAS-AFHAQFVQALTGGG

Rv0832 LSARAA-AFHHEFVQALAAGA

Rv2162c LSAQLA-AYHNQFVRALNAGA

Rv2490c LSAQAA-AFHDQFVHTLTAAA

Rv1087 VSAQMS-AFHAQFMQALTGAG

Rv1068c IARQMA-AFHDQFTLALTSSA

Rv1067c LGARAA-LFHEQFVQALTGAA

Rv1840c LSAQAA-LFHEQFVHAMTAGA

Rv0578c VSAQVA-TFHDRFVQTLSAAA

Rv2615c ISAQVA-AYQQRFVLALSQAG

Rv1768 LSAQAA-MFHEQFVRALAAGG

Rv0109 LSAQAL-AFHDQFVQALNMGA

Rv3590c LSAQAA-GFHAQFVQALNAGV

Rv2098c VSAQAA-AFHDRFVQLLNAGG

Rv3508 ISAQVA-AYHQRFVQALSTGA

Rv3514 ISAQVA-AYHQRFVQALSTGA

Rv3388 LSAQAT-IFHDQFVQALTSGG

Rv3345c LSARVA-TFHEQFVRSLTAAG

Rv1450c ISTQVA-AFHDRFAQTLSAAV

Rv1452c ISTQVA-AFHDRFAQTLSAAV

Rv1441c LSAQMA-RFHQQFVQALTASV

Rv0746 ASAQAA-AVHAQFVEALSAGA

Rv3650 ASAQAA-AFHTRFIRARSRHP

Rv2591 LNAQAA-AFHHSFVQTLNAAG

Rv3511 ISAQVA-AYHQQFVQTLRTGA

Rv0532 LSAAAA-AFHDQFVRALAAGA

Rv1243c LSAQAA-AFHQQFVRALSSAA

Rv0977 LSVQVT-AFHVQFAQTLTNAG

Rv0872c LGTQAA-AFHERFIQALSTAA

Rv2853 LSTQAA-AFHSRFVQALTTAA

Rv0834c MSAQAA-AFHARFVQALAGAM

Rv2487c LSVQVA-AFHEQFVQALTAAA

Rv3507 AAAQVA-AFHSRFVQALTAGA

Rv3367 VAAQAT-ALHDQFVQALTGAG

Rv0297 ASAQAA-AFHAQVVRTLTVDA

Rv1803c LSAHAV-AFHEQFVQLMSAGA

Rv1806 VGAQAI-AMNEAFVAMLGASA

Rv1651c LNARAA-TFHAEFVSLLNGGA

Rv0978c ISAQVA-AYQQRFVLALSQAG

Rv0980c ISAQVA-AYQERFVLALSQAS

Rv0916c VSKPAA-LVHGMFVALPAATA

Rv2340c ISAQAA-LFHDRFVQILQEGA

Rv1983 VSAQAS-AFHQQFVQTLNSAS

Rv0754 AAAQAA-AYHEQFVHRLSAAA

Rv2107 QAARGT-AFHRKLVRTLANGA

Rv2519 LSVRFA-SLDQQFAQALNSAA

Rv1088 LSAQVA-AFQTLFVRTLTGGC

Rv3652 SVRGRR-RSMSGSCRPWPQVG

Rv3022.1 IAGEGV-EELGRSGVAVGESG

Rv0285 VTAEGV-EELGRAGVGVGESG

Rv1386 MAAQGV-EELGRSGVGVAESG

Rv1172c LSRQMMTAFHDQFVQALRASA

Rv2328 LSAQAS-AFHDEFVNLLKTGA

Rv1169c IARQAH-VIHESFVQTLQTSK

Rv1646 ISAQIA-GFHNQFVGALRGGA

Rv2408 ISTQMA-AFHDQLVENRTADS

Rv0335c LNNQAA-AFHGQFAQNLNVGA

Rv3097c LSAQLA-VFHERFVQALTGAA

Rv1430 LSAQAS-AFHDRFVRALSAAA

Rv0151c ISAQVA-AFHNEFTQRLAAAA

Rv0160c VVKQAA-AFHSEFTRTLEAAG

Rv0159c VLKQAA-AFHGEFTRALAAAG

Rv3812 VSNQLA-AFHTEFVRTLNRGA

Rv3872 SNASAQ-DQLHRAGEAVQDVA
