# Supplementary material for: Identifying Cognate Binding Pairs among a Large Set of Paralogs: The Case of PE/PPE Proteins of Mycobacterium tuberculosis
Source: PLoS Comput Biol. 2008 Sep 12;4(9):e1000174. doi: 10.1371/journal.pcbi.1000174 (PMC2519833; doi:10.1371/journal.pcbi.1000174)
Supplement: Dataset S2 — Structure-based alignment of PPE proteins. (0.05 MB DOC) [file pcbi.1000174.s002.doc]

CLUSTAL W (1.83) multiple sequence alignment

!SS_Rv2430c ......aaaAAAAaaa....aaaAAAAAAAAAAAAAAAAAAAAAAaaa....-....---

Rv2430c HFEAYPPEVNSANIYAGPGPDSMLAAARAWRSLDVEMTAVQRSFNRTLLSLM-DAWA---

Rv0388c DFGALPPEINSARIYSGPGSRPLMQAAAAWQRLANELTATAASYSSVISGLTGDDWL---

Rv1808 DFGALPPEINSGRMYAGPGSGPLLAAAAAWDALAAELYSAAASYGSTIEGLTVAPWM---

Rv3135 DYAFLPPEINSARMYSGPGPNSMLVAAASWDALAAELASAAENYGSVIARLTGMHWW---

Rv3532 DFAMLPPEVNSTRMYSGPGAGSLWAAAAAWDQVSAELQSAAETYRSVIASLTGWQWL---

Rv1801 DFGLLPPEINSGRMYTGPGPGPMLAAATAWDGLAVELHATAAGYASELSALT-GAWS---

Rv3136 DFALLPPEVNSARMYTGPGAGSLLAAAGGWDSLAAELATTAEAYGSVLSGLAALHWR---

Rv1802 DFGVLPPEINSGRMYAGPGSGPMLAAAAAWDGLATELQSTAADYGSVISVLT-GVWS---

Rv1807 DFATLPPEINSARMYSGAGSAPMLAAASAWHGLSAELRASALSYSSVLSTLTGEEWH---

Rv1809 DFGLQPPEITSGEMYLGPGAGPMLAAAVAWDGLAAELQSMAASYASIVEGMASESWL---

Rv1789 DFGALPPEVNSVRMYAGPGSAPMVAAASAWNGLAAELSSAATGYETVITQLSSEGWL---

Rv1705c DFGALPPEVNSGRMYCGPGSAPMVAAASAWNGLAAELSVAAVGYERVITTLQTEEWL---

Rv1790 DFGALPPEINSGRMYCGPGSGPMLAAAAAWDGVAVELGLAATGYASVIAELTGAPWV---

Rv1787 DFGALPPEINSGRMYCGPGSGPMLAAAAAWDGVAVELGLAATGYASVIAELTGAPWV---

Rv2892c DFGVLPPEINSGRMYAGPGSGPMMAAAAAWDSLAAELGLAAGGYRLAISELTGAYWA---

Rv2768c DFGALPPEINSTRMYAGAGAAPLMAAGATWNGLAVELSTTASSVESVIMQLTTEQWL---

Rv1039c DFGALPPEINSARMYAGAGAGPMMAAGAAWNGLAAELGTTAASYESVITRLTTESWM---

Rv2770c DFGALPPEVNSARMYGGAGAADLLAAAAAWNGIAVEVSTAASSVGSVITRLSTEHWM---

Rv3621c DFAQLPPEVNSALMYAGPGSGPMLAAAAAWEALAAELQTTASTYDALITGLADGPWQ---

Rv1706c NFGALPPEINSGRMYSGPGSGPLMAAAAAWDGLAAELSSAATGYGAAISELTNMRWWS--

Rv1361c DFGALPPEINSARMYAGPGSASLVAAAKMWDSVASDLFSAASAFQSVVWGLTTGSWI---

Rv3478 DFGALPPEINSARMYAGPGSASLVAAAKMWDSVASDLFSAASAFQSVVWGLTVGSWI---

Rv1196 DFGALPPEINSARMYAGPGSASLVAAAQMWDSVASDLFSAASAFQSVVWGLTVGSWI---

Rv3739c IWFASPPEVHSALLSAGPGPASLQAAAAEWTSLSAEYASAAQELTAVLAAVQGGAWE---

Rv3018c VWLASPPEVHSALLSAGPGPGSLQAAAAGWSALSAEYAAVAQELSVVVAAVGAGVWQ---

Rv3022c VWLASPPEVHSALLSAGPGPGSLQAAAAGWSALSAEYAAVAQELSVVVAAVGAGVWQ---

Rv0286 IWMASPPEVHSALLSNGPGPGSLVAAATAWSQLSAEYASTAAELSGLLGAVPGWAWQ---

Rv0256c IWMASPPEVHSALLSSGPGPGPLLVSAEGWHSLSIAYAETADELAALLAAVQAGTWD---

Rv2123 MWFAVPPEVPSAWLSTGMGPGPLLAAARAWHALAAQYTEIATELASVLAAVQASSWQ---

Rv0453 IWMASPPEVHSALLSSGPGPGPVLAAATGWSSLGREYAAVAEELGALLAAVQAGVWQ---

Rv0280 LWMASPPEVHSALLSSGPGPGSVLSAAGVWSSLSAEYAAVADELIGLLGAVQTGAWQ---

Rv0096 --MAIPPEVHSGLLSAGCGPGSLLVAAQQWQELSDQYALACAELGQLLGEVQASSWQ---

Rv1387 PWIAFPPEVHSAMLNYGAGVGPMLISATQNGELSAQYAEAASEVEELLGVVASEGWQ---

Rv0915c DFGLLPPEVNSSRMYSGPGPESMLAAAAAWDGVAAELTSAAVSYGSVVSTLIVEPWM---

Rv3738c ------------------------------------------------------------

Rv2352c DFSWLPPEINSARIYAGAGSGPLFMAAAAWEGLAADLRASASSFDAVIAGLAAGPWS---

Rv3125c GFSWLPPEINSARMFAGAGSGPLFAAASAWEGLAADLWASASSFESVLAALTTGPWT---

Rv3350c EFPVLPPEINSVLMYSGAGSSPLLAAAAAWDGLAEELGSAAVSFGQVTSGLTAGVWQ---

Rv3347c NFPVLPPEINSVLMYSGAGSSPLLAAAAAWDGLAEELGSAAVSFGQVTSGLTAGVWQ---

Rv3343c SFVVMPPEINSLLIYTGAGPGPLLAAAAAWDELAAELGSAAAAFGSVTSGLVGGIWQ---

Rv0355c SFAVLPPEINSARLYVGAGLAPMLDAAAAWDGLADELGSAAASFSAVTAGLAGSSWL---

Rv3533c NYAVLPPELNSLRMFTGAGSAPMLAAAVAWDGLAAELGSAASSFGSVTSDLASQAWQ---

Rv3159c NYSVLPPEINSLRMFTGAGSAPMLAASVAWDRLAAELAVAASSFGSVTSGLAGQSWQ---

Rv1135c -FLVLPPEVNSALMFAGAGSGPTLAAAAAWDGLAAELGQAANSFSSATAALADTAWQ---

Rv2356c NFSVLPPEINSGRMFFGAGSGPMLAAAAAWDGLAAELGLAAESFGLVTSGLAGGSGQAWQ

Rv3558 HFSVLPPEINSLRMYLGAGSAPMLQAAAAWDGLAAELGTAASSFSSVTTGLTGQAWQ---

Rv0755c GFAWLPPETNSLRMYLGAGSRPLLAAAGAWDGLAEELHAAASSFGSVTSELAGGAWQ---

Rv1548c NFSVLPPEINSALMFAGAGPGPMLAAASAWTGLAGDLGSAAASFSAVTSQLATGSWQ---

Rv0878c NFMVLPPEVNSARIYAGAGPAPMLAAAVAWDGLAAELGMAAASFSLLISGLTAGPGSAWQ

Rv0305c DFVVSAPEVNSLRMYLGAGSGPMLAAAAAWDGLADELAVAASWFGSVTSGLADAAWR---

Rv1917c NFSTLPPEINSALIFGGAGSEPMSAAAVAWDQLAMELASAAASFNSVTSGLVGESWL---

Rv0442c HFAWLPPEINSALMFAGPGSGPLIAAATAWGELAEELLASIASLGSVTSELTSGAWL---

Rv1753c NFSVLPPEINSALIFAGAGPEPMAAAATAWDGLAMELASAAASFGSVTSGLVGGAWQ---

Rv1918c HYSVLPPEINSALIFAGAGSGPMLAAASAWDGLATELASAAVSFGSVTAGLVGGSWQ---

Rv3144c SFVVLPPEINSLRMFIGAGTAPMLAAAAAWDGLAEELGTAAQSFASVTAGLAGQAWQ---

Rv3873 LWHAMPPELNTARLMAGAGPAPMLAAAAGWQTLSAALDAQAVELTARLNSLGEAWT----

Rv1168c DFTIFPPEFNSLNIQG--SARPFLVAANAWKNLSNELSYAASRFESEINGLITSWRG---

Rv2108 NFWALPPEINSTRIYLGPGSGPILAAAQGWNALASELEKTKVGLQSALDTLLESYRG---

Rv3425 MHPMIPAEYISNIIYEGPGADSLFFASGQLRELAYSVETTAESLEDELDELDENWK----

Rv3429 MHPMIPAEYISNIIYEGPGADSLSAAAEQLRLMYNSANMTAKSLTDRLGELQENWK----

Rv3426 MHLMIPAEYISNVIYEGPRADSLYAADQRLRQLADSVRTTAESLNTTLDELHENWK----

Rv2608 NFAVLPPEVNSARIFAGAGLGPMLAAASAWDGLAEELHAAAGSFASVTTGLAGDAWH---

Rv1800 NFAVLPPEVNSARVFAGAGSAPMLAAAAAWDDLASELHCAAMSFGSVTSGLVVGWWQ---

Rv3539 DFLTLSPEVNSARMYAGGGPGSLSAAAAAWDELAAELWLAAASFESVCSGLADRWWQ---

Rv3892c GWAARTPEANDLLLTAGTGVGTHLANQTAWTTLGASHHASGVASAINTAATAASWLG---

!SS_Rv2430c .aaaAAAaaa.aaaAAAAAAAAAAAAAAAAAAAAAAAAAAAAaaa...aaaAAAAAAAAA

Rv2430c GPVVMQLMEAAKPFVRWLTDLCVQLSEVERQIHEIVRAYEWAHHDMVPLAQIYNNRAERQ

Rv0388c GPSALSMAAAAVPYVAWMRATAASAEQAAAQAVAAANAYESAYAATVPPTVIAANRRTML

Rv1808 GPSSITMAAAVAPYVAWISVTAGQAEQAGAQAKIAAGVYETAFAATVPPPVIEANRALLM

Rv3135 GPASTSMLAMSAPYVEWLERTAAQTKQTATQARAAAAAFEQAHAMTVPPALVTGIRGAIV

Rv3532 GPSSVRMGAAVTPYVEWLTTTAAQARQTATQITAAATGFEQAFAMTVPPPAIMANRAQVL

Rv1801 GPSSTSMASAAAPYVAWMSATAVHAELAGAQARLAIAAYEAAFAATVPPPVIAANRAQLM

Rv3136 GPAAESMAVTAAPYIGWLYTTAEKTQQTAIQARAAALAFEQAYAMTLPPPVVAANRIQLL

Rv1802 GQSSGTMAAAAAPYVAWMSATAALAREAAAQASAAAAAYEAAFAATVPPPVVAANRAELA

Rv1807 GPASASMTAAAAPYVAWMSVTAVRAEQAGAQAEAAAAAYEAAFAATVPPPVIEANRAQLM

Rv1809 GPSSAGMAAAAAPYVTWMSGTSAQAKAAADQARAAVVAYETAFAAVVPPPQIAANRSQLI

Rv1789 GPASAAMAEAVAPYVAWMSAAAAQAEQAATQARAAAAAFEAAFAATVPPPLIAANRASLM

Rv1705c GPASTLMVEAVAPYVAWMRATAIQAEQAASQARAAAAAYETAFAAIVPPPLIAANRARLT

Rv1790 GAASLSMVAAATPYVAWLSQAAARAEQAGMQAAAAAAAYEAAFVMTVPPPVITANRVLVM

Rv1787 GAASLSMVAAATPYVAWLSQAAARAEQAGMQAAAAAAAYEAAFVMTVPPPVITANRVLVM

Rv2892c GPAAASMVAAVTPYVAWLSATAGQAEQAGMQARAAAAAYELAFAMTVPPPVVVANRALLV

Rv2768c GPASMSMVVAAQPYLAWLTYTAESAAHAAAQAMASAAAFEAAFAMTVPPAEVAANRALLA

Rv1039c GPASMAMVAAAQPYLAWLTYTAEAAAHAGSQAMASAAAYEAAYAMTVPPEVVAANRALLA

Rv2770c GPASLSMAAAVQPYLVWLTCTAESSALAAAQAMASAAAFETAFALTVPPAEVVANRALLA

Rv3621c GSSAASMVAAATPQVAWLRSTAGQAEQAGSQAVAAASAYEAAFFATVPPPEIAANRALLM

Rv1706c GPASDSMVAAVLPFVGWLSTTATLAEQAAMQARAAAAAFEAAFAMTVPPPAIAANRTLLM

Rv1361c GSSAGLMVAAASPYVAWMSVTAGQAELTAAQVRVAAAAYETAYGLTVPPPVIAENRAELM

Rv3478 GSSAGLMAAAASPYVAWMSVTAGQAQLTAAQVRVAAAAYETAYRLTVPPPVIAENRTELM

Rv1196 GSSAGLMVAAASPYVAWMSVTAGQAELTAAQVRVAAAAYETAYGLTVPPPVIAENRAELM

Rv3739c GPSAEAYVAAHLPYLA--------------------------------------------

Rv3018c GPSAELFVAAYVPYVAWLVQASADSAAAAGEHEAAAAGYVCALAEMPTLPELAANHLTHA

Rv3022c GPSAELFVAAYVPYVAWLVQASADSAAAAGEHEAAAAGYVCALAEMPTLPELAANHLTHA

Rv0286 GPSAEWYVAAHLPYVAWLTQASADAAGAAAQHEAAAAAYTTALAAMPTLAELAANHVIHT

Rv0256c GPTAAVYVAAHTPYLAWLVQASANSAAMATRQETAATAYGTALAAMPTLAELGANHALHG

Rv2123 GPSADRFVVAHQPFRYWLTHAATVATAAAAAHETAAAGYTSALGGMPTLAELAANHAMHG

Rv0453 GPSAESFAAACLPYLSWLTQASADCAAAAARLEAVTAAYAAALVAMPTLAELAANHATHG

Rv0280 GPSAAAYVAAHAPYLAWLMRASETSAEAAARHETVAAAYTTAVAAMPTLVELAANHTLHG

Rv0096 GTAATQYVAAHGPYLAWLEQTAINSAVTAAQHVAAAAAYCSALAAMPTPAELAANHAIHG

Rv1387 GQAAEAFVAAYMPFLAWLIQASADCVEMAAQQHVVIEAYTAAVELMPTQVELAANQIKLA

Rv0915c GPAAAAMAAAATPYVGWLAATAALAKETATQARAAAEAFGTAFAMTVPPSLVAANRSRLM

Rv3738c ----------------------------------MTTAYASALAAMPTLTELAANHTSHA

Rv2352c GPASVAMAGAAAPYVGWLSAAAGQAELSAGQATAAATAFEAALAATVHPAAVTANRVLLG

Rv3125c GPASMSMAAAASPYVGWLSTVASQAQLAAIQARAAATAFEAALAATVHPTAVTANRVSLA

Rv3350c GAAAAAMAAAAAPYAGWLGSVAAAAEAVAGQARVVVGVFEAALAATVDPALVAANRARLV

Rv3347c GAAAAAMAAAAAPYAGWLGSVAAQAVAVAGQARAAVAAFEAALAATVDPAAVAVNRMAMR

Rv3343c GPSSVAMAAAAAPYAGWLSAAAASAESAAGQARAVVGVFEAALAETVDPFVIAANRSRLV

Rv0355c GAASTAMTGAAAPYLGWLSAAAAQAQQAATQTRLAAAAFEAALAATVHPAIISANRALFV

Rv3533c GPAAAAMAAAAAPYAGWLSAAAARAAGAAAQAKAVASAFEAARAATVHPLLVAANRNAFA

Rv3159c GAAAAAMAAAAAPYAGWLAAAAARAAGASAQAKAVASAFEAARAATVHPMLVAANRNAFV

Rv1135c GPAATAMAAAAAPYASWLSTAATRALSAAAQAKAAAAVYEAARAATVDPLLVAANRHQLV

Rv2356c GAAAAAMVVAAAPYAGWLAAAAARAGGAAVQAKAVAGAFEAARAAMVDPVVVAANRSAFV

Rv3558 GPASAAMAAAAAPYAGFLTTASAQAQLAAGQAKAVASVFEAAKAAIVPPAAVAANREAFL

Rv0755c GPASAAMANAAGPYASWLTAAGAQAELAARQARAAAGAFEEALAGVVHPAVVQANRVRTW

Rv1548c GPASAAMTGVAASYARWLTTAAAQAEQAAGQAQAAVSAFEAALAATVHPGAVSANRGRLR

Rv0878c GPAAAAMAAAAAPYLSWLNAATARAEGAAAGAKAAAAVYEAARAATAHPALVAANRNQLL

Rv0305c GPAAVAMARAVAPYLGWLISATAQAEQAAAQARVAVATFEAARAATVHPAIVAANRAVLV

Rv1917c GPSSAAMAAAVAPYLGWLAAAAAQAQRSATQAAALVAEFEAVRAAMVQPALVAANRSDLV

Rv0442c GPSAAAMMAVATQYLAWLSTAAAQAEQAAAQAMAIATAFEAALAATVQPAVVAANRGLMQ

Rv1753c GASSSAMAAAAAPYAAWLAAAAVQAEQTAAQAAAMIAEFEAVKTAVVQPMLVAANRADLV

Rv1918c GRSSVAMAAAAAPYAGWLAAAATQAEQAATQAQVMVAEFEAVRLAMVQPALVAANRSGLI

Rv3144c GPAALAMAAAAAPYAGWLTAAAAQSAGAAGQARAVASIFEAAQAATVLPAAVAANRDAFV

Rv3873 GGGSDKALAAATPMVVWLQTASTQAKTRAMQATAQAAAYTQAMATTPSLPEIAANHITQA

Rv1168c -PSSTIMAAAVAPFRAWIVTTASLAELVADHISVVAGAYEAAHAAHVPLPVIETNRLTRL

Rv2108 -QSSQALIQQTLPYVQWLTTTAEHAHKTAIQLTAAANAYEQARAAMVPPAMVRANRVQTT

Rv3425 GSSSDLLADAVERYLQWLSKHSSQLKHAAWVINGLANAYNDTRRKVVPPEEIAANREERR

Rv3429 GSSSDLMADAAGRYLDWLTKHSRQILETAYVIDFLAYVYEETRHKVVPPATIANNREEVH

Rv3426 GSSSEWMADAALRYLDWLSKHSRQILRTARVIESLVMAYEETLLRVVPPATIANNREEVR

Rv2608 GPASLAMTRAASPYVGWLNTAAGQAAQAAGQARLAASAFEATLAATVSPAMVAANRTRLA

Rv1800 GSASAAMVDAAASYIGWLSTSAAHAEGAAGLARAAVSVFEEALAATVHPAMVAANRAQVA

Rv3539 GPSSRMMAAQAARHTGWLAAAATQAEGAASQAQTMALAYEAAFAATVHPALVAANRALVA

Rv3892c -VGSAASALNVTMLNATLHGLAGWVDVKPAVVSTAIAAFETANAAMRPAPECMENRDEWG

!SS_Rv2430c Aaaa.....----..aaaAAAAAAAAAAAAAAAAAAAAAAAAAAAAaaa..-...-----

Rv2430c ILIDNNALG----QFTAQIADLDQEYDDFWDEDGEVMRDYRLRVSDALSKL-TPW-----

Rv0388c SLVQTNVFG----QNTPAIATSETHYGEMWAHDILAMDGYAGASGAASQLR---------

Rv1808 SLVATNIFG----QNTPAIAATEAHYAEMWAQDAAAMYGYAGSSATASQLA-PFSEPPQT

Rv3135 VETASASNT----AGTPP------------------------------------------

Rv3532 SLIATNFFG----QNTAAIAALETQYAEMWEQDATAMYDYAATSAAARTLT-PFTSPQQD

Rv1801 VLIATNIFG----QNTPAIMMTEAQYMEMWAQDAAAMYGYAGSSATASRMT-AFTEPPQT

Rv3136 ALIATNFFG----QNTAAIAATEAQYAEMWAQDAAAMYGYATASAAAALLT-PFSPPRQT

Rv1802 VLAATNIFG----QNTGAIAAAEARYAEMWAQDAAAMYGYAGSSSVATQVT-PFAAPPPT

Rv1807 ALIATNVLG----QNAPAIAATEAQYAEMWSQDAMAMYGYAGASAAATQLT-PFTEPVQT

Rv1809 SLVATNIFG----QNTAAIAATEAEYGEMWAQDTMAMFGYASSSATASRLT-PFTAPPQT

Rv1789 QLISTNVFG----QNTSAIAAAEAQYGEMWAQDSAAMYAYAGSSASASAVT-PFSTPPQI

Rv1705c SLVTHNVFG----QNTASIAATEAQYAEMWAQDAMAMYGYAGSSATATKVT-PFAPPPNT

Rv1790 TLIATNFFG----QNSAAIAVAEAQYAEMWAQDAVAMYGYAAASASASRLI-PFAAPPKT

Rv1787 TLIATNFFG----QNSAAIAVAEAQYAEMWAQDAVAMYGYAAASASASRLI-PFAAPPKT

Rv2892c ALVATNFFG----QNTPAIAATEAQYAEMWAQDAAAMYAYAGSAAIATELT-PFTAAPVT

Rv2768c ALVATNVLG----QNTPAIMATEAHYGEMWAQDALAMYGYAASSAAAGRLN-PLITPSQT

Rv1039c ALVATNVLG----INTPAIMATEALYAEMWAQDALAMYGYAAASGAAGMLQ-PLSPPSQT

Rv2770c ELTATNILG----QNVSAIAATEARYGEMWAQDASAMYGYAAASAVAARLN-PLTRPSHI

Rv3621c ALLATNFLG----QNTAAIAATEAQYAEMWAQDAAAMYGYAGASAAATQLS-PFNPAAQT

Rv1706c TLVDTNWFG----QNTPAIATTESQYAEMWAQDAAAMYGYASAAAPATVLT-PFAPPPQT

Rv1361c ILIATNLLG----QNTPAIAVNEAEYGEMWAQDAAAMFGYAATAATATEALLPFEDAPLI

Rv3478 TLTATNLLG----QNTPAIEANQAAYSQMWGQDAEAMYGYAATAATATEALLPFEDAPLI

Rv1196 ILIATNLLG----QNTPAIAVNEAEYGEMWAQDAAAMFGYAAATATATATLLPFEEAPEM

Rv3739c ------------------------------------------------------------

Rv3018c VLVATNFFG----INTIPIALNEADYVRMWVQAATVMSAYEAVVGAALVATPHTGPAPVI

Rv3022c VLVATNFFG----INTIPIALNEADYVRMWVQAATVMSAYEAVVGAALVATPHTGPAPVI

Rv0286 VLVATNFFG----INTIPITLNEADYVRMWLQAAAVMGLYQAASGAALASAPRTVPAPTV

Rv0256c VLMATNFFG----INTIPIALNESDYARMWIQAATTMASYQAVSTAAVAAAPQTTPAPQI

Rv2123 ALVTTNFFG----VNTIPIALNEADYLRMWIQAATVMSHYQAVAHESVAATPSTPPAPQI

Rv0453 AMVATNFFG----INTIPIAVNEADYVRMWLQAATTMATYQAVADSAVRSIPDSVPPPRI

Rv0280 VLVATNFFG----INTIPIALNEADYARMWTQAASTMATYQAVAEAAVASAPQTTPAPPI

Rv0096 VLIATNFFG----INTVPIALNEADYVRMWLQAADTMAAYQAVADAATVAVPSTQPAPPI

Rv1387 VLVATNFFG----INTIPIAINEAEYVEMWVRAATTMATYSTVSRSALSAMPHTSPPPLI

Rv0915c SLVAANILG----QNSAAIAATQAEYAEMWAQDAAVMYSYEGASAAASALP-PFTPPVQG

Rv3738c VLLGTNFFG----INTIPIALNEADYARMWIQAATTMSIYEGTSDAALASAPQTTPAPVL

Rv2352c ALVATNILG----QNTPAIAATEFDYVEMWAQDVGAMVGYHAGAAAVAETLTPFSVPP--

Rv3125c SLIAANVLG----QNTPAIAATEFDYLEMWAQDVAAMVGYHAGAKSVAATLAPFSLPP--

Rv3350c ALAVSNLLG----QNTPAIAAAEAEYELMWAADVAAMAGYHSGASAAAAALPAFSPPAQA

Rv3347c ALAMSNLLG----QNAAAIAAVEAEYELMWAADVAAMAGYHSGASAAAAALPAFSPPAQA

Rv3343c SLALSNLFG----QNTPAIAAAEFDYELMWAQDVAAMLGYHTGASAAAEALAPFGSPLAS

Rv0355c SLVVSNLLG----QNAPAIAATEAAYEQMWAQDVAAMFGYHAGASAAVSALTPFGQALPT

Rv3533c QLVMSNWFG----LNAPLIAAVEGAYEQMWAADVAAMVGYHSGASAAAEQLVPFQQALQQ

Rv3159c QLVLSNLFG----QNAPAIAAAEAMYEQMWAADVAAMVGYHGGASAAAAQLSSWSIGLQQ

Rv1135c SLVLSNLFG----QNAPAIAATEAAYEQLWAADVAAMVSYHSGASAVAAQLAPWAQAVRA

Rv2356c QLVLSNVFG----QNAPAIAAAEATYEQMWAADVAAMVGYHGGASAAAAALAPWQQAVPG

Rv3558 ALIRSNWLG----LNAPWIAAVESLYEEYWAADVAAMTGYHAGASQAAAQLPLPAGLQQF

Rv0755c LLAVSNVFG----QNAPAIAAMESTYEQMWAQDVAVMAGYHAASSAAAAQLASWQPALPN

Rv1548c SLVASNLLG----QNAPAIAAVEAVYEQMWAADVAAMLGYHGEASAVALSLTPFTPSPSA

Rv0878c SLVLSNLFG----QNLPAIAATEASYEQLWAQDVAAMVGYHGGASTVASQLTPWQQLLSV

Rv0305c SLVSSNLLG----FNAPAIAATEAAYERMWAQDVAAMVGYHAGASAAVSALMPFTQQLKK

Rv1917c SLVFSNFFG----QNAPAIAAIEAAYEQMWAIDVSVMSAYHAGASAVASALTPFTAPPQN

Rv0442c LLAATNWFG----QNAPALMDVEAAYEQMWALDVAAMAGYHFDASAAVAQLAPWQQVLRN

Rv1753c SLVMSNLFG----QNAPAIAAIEATYEQMWAADVSAMSAYHAGASAIASALSPFSKPLQN

Rv1918c SLVISNLFG----QNAPAIAAAEAAYEEMWALDVSAMAAYHSGASAVAVALPAFALPLRL

Rv3144c QLVMTNLFG----QNAPLIAAAEGVYEEMWAADVAAMSGYYSGASAIAAQVVPWASLLQR

Rv3873 VLTATNFFG----INTIPIALTEMDYFIRMWNQAALAMEVYQAETAVNTLFEKLEPMASI

Rv1168c ALATTNIFG----IHTPAIFALDALYAQYWSQDGEAMNLYATMAAAAARLT-PFSPPAPI

Rv2108 VLKAINWFG----QFSTRIADKEADYEQMWFQDALVMENYWEAVQEAIQSTSHFEDPPEM

Rv3425 RLIASNVAG----VNTPAIADLDAQYDQYRARNVAVMNAYVSWTRSALSDLPRWREPPQI

Rv3429 RLIASNVAG----VNTPAIAGLDAQYQQYRAQNIAVMNDYQSTARFILAYLPRWQEPPQI

Rv3426 RLIASNVAGGKHSSNRRPRGTIRAVPGRKYPSNGPLSKLDPICAIEAAPMAGAAADPQER

Rv2608 SLVAANLLG----QNAPAIAAAEAEYEQIWAQDVAAMFGYHSAASAVATQLAPIQEGLQQ

Rv1800 SLVASNLFG----QNAPAIAALESLYECMWAQDAAAMAGYYVGASAVATQLASWLQRLQS

Rv3539 WLAGSNVFG----QNTPAIAAAEAIYEQMWAQDVVAMLNYHAVASAVGARLRPWQQLLHE

Rv3892c VDNAINPSVL--WTLTPRIVSLDVEYFGVMWPNNAAVGATYGGVLAALAESLAIPPPVAT

!SS_Rv2430c .......

Rv2430c KAPPPIA

Rv0388c RSPATGD

Rv1808 TNPSATA

Rv3135 -------

Rv3532 TNSAGLP

Rv1801 TNHGQLG

Rv3136 TNPAGLT

Rv1802 TNAAGLA

Rv1807 TNASGLA

Rv1809 TNPSGLA

Rv1789 ANPTAQG

Rv1705c TSPSAAA

Rv1790 TNSAGVV

Rv1787 TNSAGVV

Rv2892c TSPAALA

Rv2768c ANMAGLA

Rv1039c TNPGGLA

Rv2770c TNPAGLA

Rv3621c INPAGLA

Rv1706c TNATGLV

Rv1361c TNPGGLL

Rv3478 TNPGGLL

Rv1196 TSAGGLL

Rv3739c -------

Rv3018c VKPGANE

Rv3022c VKPGANE

Rv0286 MNPGGGA

Rv0256c VKANAPT

Rv2123 VTSAASS

Rv0453 LKSNAQS

Rv0280 LAAEAAD

Rv0096 RAPGGDA

Rv1387 LKSDELL

Rv0915c TGPAGPA

Rv3738c FNGGAGV

Rv2352c LDLAGLA

Rv3125c VSLAGLA

Rv3350c LGGGVGA

Rv3347c LGGGVGA

Rv3343c LAAAAEP

Rv0355c VAGGGAL

Rv3533c LPNLGIG

Rv3159c ALPAAPS

Rv1135c LPNPTAP

Rv2356c LSGLLGG

Rv3558 LNTLPNL

Rv0755c INLGVGN

Rv1548c AATPGGA

Rv0878c LPPVVTA

Rv0305c LAGLSER

Rv1917c LTDLPAQ

Rv0442c LGIDIGK

Rv1753c LAGLPAW

Rv1918c PAGLAAG

Rv3144c FPGLGAG

Rv3873 LDPGASQ

Rv1168c ANPGALA

Rv2108 ADDYDEA

Rv3425 YRGG---

Rv3429 YGGGGG-

Rv3426 VGPRGRR

Rv2608 QLQNVLA

Rv1800 IPGAASL

Rv3539 LPRRLGG

Rv3892c MGASPAA
