# Supplementary figures and images for: Identifying Cognate Binding Pairs among a Large Set of Paralogs: The Case of PE/PPE Proteins of Mycobacterium tuberculosis
Source: PLoS Comput Biol. 2008 Sep 12;4(9):e1000174. doi: 10.1371/journal.pcbi.1000174 (PMC2519833; doi:10.1371/journal.pcbi.1000174)

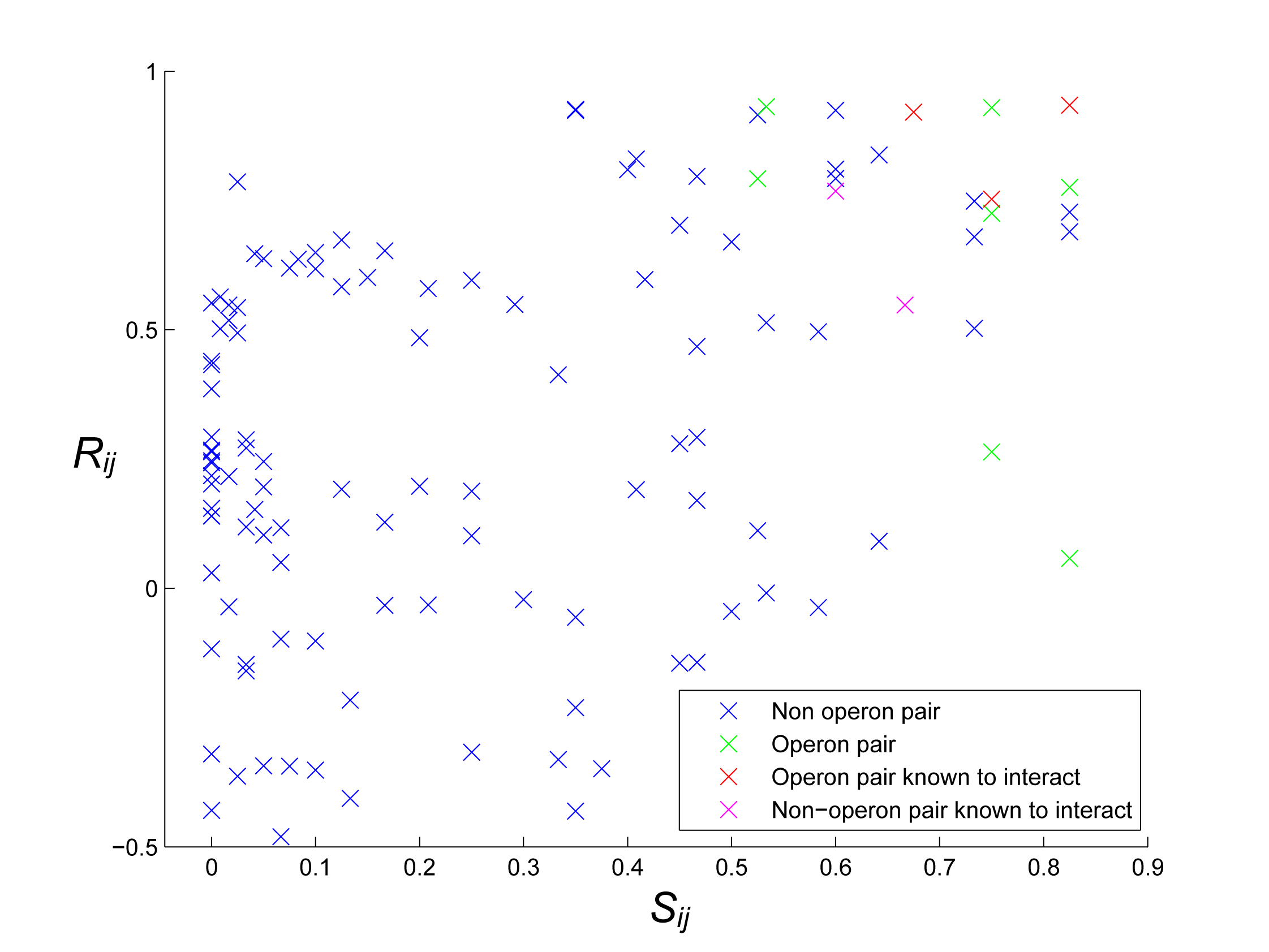

Supplement: Figure S1 — Predicted interactions of ESAT-6/CFP-10 (Esx) family proteins. Interactions are scored by Sij and Rij. Esx pairs not in operons are shown as blue crosses; operon pairs are green; operon pairs found in experiments to interact are red; non-operon pairs found to interact are magenta. Notice that all known interactions (red and magenta) and nearly all operon pairs (green) tend to be highly scored by our method (tending towards the upper right of the plot), suggesting our method's applicability to other protein families. (11.34 MB TIF) [file pcbi.1000174.s006.tif]

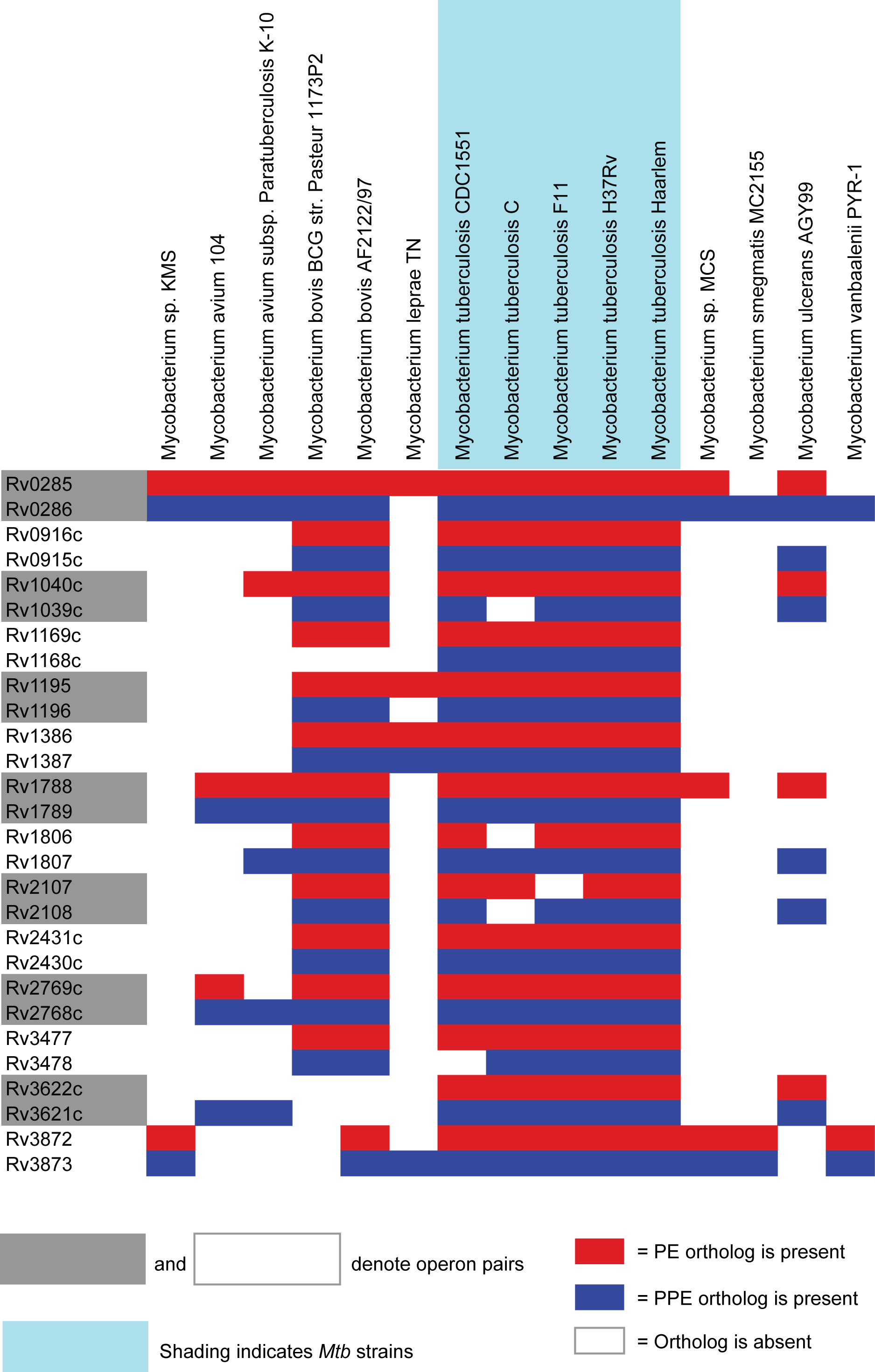

Supplement: Figure S2 — Comparative genomic analysis of PE/PPE operon pairs. The ORF identifiers of PE/PPE operon pairs from H37Rv are shown on the left on alternating gray and white backgrounds. Genomes containing orthologous operon pairs are shown at the top, with Mtb genomes shaded in cyan. Red rectangles show the presence of PEs; blue show PPEs. White spaces indicate that no ortholog was found in that genome. Notice that the PE/PPE pairs appear well-conserved in Mtb and M. bovis strains, and that a few operon pairs are disrupted by the loss of a gene in the Mtb CDC1551, C, and F11 strains. (16.58 MB TIF) [file pcbi.1000174.s007.tif]
