# Supplementary material for: Identifying Cognate Binding Pairs among a Large Set of Paralogs: The Case of PE/PPE Proteins of Mycobacterium tuberculosis
Source: PLoS Comput Biol. 2008 Sep 12;4(9):e1000174. doi: 10.1371/journal.pcbi.1000174 (PMC2519833; doi:10.1371/journal.pcbi.1000174)
Supplement: Table S1 — Gene expression datasets used. (0.04 MB DOC) [file pcbi.1000174.s008.doc]

| **GEO accession** | **Experimental conditions probed** | **Number of experiments** |
| --- | --- | --- |
| GSE1642 | Inhibitors of metabolism | 437 |
| GSE3201 | *Mtb* clinical isolates, growth conditions | 48 |
| GSE365 | Mitomycin C, UV irradiation, hydrogen peroxide | 28 |
| GSE5977 | Exponential growth phase and late log phase | 6 |
| GSE10336 | Hypoxia | 2 |
| GSE6209 | Infection of human macrophages | 11 |
| GSE8786 | Stationary phase and low-oxygen dormancy | 54 |
| GSE8839 | Nitric oxide | 131 |
| GSE9776 | Various dormancy models | 17 |
|  |  |  |
|  | Total = | 734 |

**Table S1**. Gene expression datasets used.
