# Supplementary material for: Identifying Cognate Binding Pairs among a Large Set of Paralogs: The Case of PE/PPE Proteins of Mycobacterium tuberculosis
Source: PLoS Comput Biol. 2008 Sep 12;4(9):e1000174. doi: 10.1371/journal.pcbi.1000174 (PMC2519833; doi:10.1371/journal.pcbi.1000174)
Supplement: Text S1 — Accession numbers. ORF identifiers, gene names, and SwissProt accession codes for proteins analyzed in this study. (0.14 MB DOC) [file pcbi.1000174.s009.doc]

**Protein Accession Codes**

| **ORF** | **Gene name** | **SwissProt accession** |
| --- | --- | --- |
| Rv0096 | PPE1 | Q10892 |
| Rv0109 | PE_PGRS1 | Q79G09 |
| Rv0124 | PE_PGRS2 | Q79G08 |
| Rv0151c | PE1 | Q79G06 |
| Rv0159c | PE3 | Q79G04 |
| Rv0160c | PE4 | Q7DAC9 |
| Rv0256c | PPE2 | Q79FZ4 |
| Rv0278c | PE_PGRS3 | P56877 |
| Rv0279c | PE_PGRS4 | Q79FY9 |
| Rv0280 | PPE3 | Q79FY8 |
| Rv0285 | PE5 | Q7DA36 |
| Rv0286 | PPE4 | Q7DA35 |
| Rv0297 | PE_PGRS5 | Q6MX50 |
| Rv0305c | PPE6 | Q6MX48 |
| Rv0335c | PE6 | O86338 |
| Rv0355c | PPE8 | Q6MX44 |
| Rv0388c | PPE9 | Q6MX42 |
| Rv0442c | PPE10 | P42611 |
| Rv0453 | PPE11 | Q6MX41 |
| Rv0532 | PE_PGRS6 | Q6MX30 |
| Rv0578c | PE_PGRS7 | Q6MX28 |
| Rv0742 | PE_PGRS8 | Q79FW9 |
| Rv0746 | PE_PGRS9 | Q79FW8 |
| Rv0747 | PE_PGRS10 | O53810 |
| Rv0754 | PE_PGRS11 | Q79FW5 |
| Rv0755c | PPE12 | Q79FW4 |
| Rv0832 | PE_PGRS12 | Q79FV8 |
| Rv0834c | PE_PGRS14 | Q79FV6 |
| Rv0872c | PE_PGRS15 | Q79FV3 |
| Rv0878c | PPE13 | Q10540 |
| Rv0915c | PPE14 | Q79FV1 |
| Rv0916c | PE7 | Q7D937 |
| Rv0977 | PE_PGRS16 | Q79FU3 |
| Rv0978c | PE_PGRS17 | Q79FU2 |
| Rv0980c | PE_PGRS18 | Q79FU0 |
| Rv1039c | PPE15 | Q7D8Y7 |
| Rv1040c | PE8 | Q7D8Y6 |
| Rv1067c | PE_PGRS19 | Q79FT3 |
| Rv1068c | PE_PGRS20 | O53416 |
| Rv1087 | PE_PGRS21 | Q79FT0 |
| Rv1088 | PE9 | Q79FS8 |
| Rv1091 | PE_PGRS22 | Q79FS5 |
| Rv1135c | PPE16 | Q79FS0 |
| Rv1168c | PPE17 | Q7D8Q2 |
| Rv1169c | PE11 | Q79FR5 |
| Rv1172c | PE12 | Q7D8P8 |
| Rv1195 | PE13 | Q79FR3 |
| Rv1196 | PPE18 | Q7D8M9 |
| Rv1214c | PE14 | Q7D8L5 |
| Rv1243c | PE_PGRS23 | Q79FQ7 |
| Rv1325c | PE_PGRS24 | Q10637 |
| Rv1361c | PPE19 | Q11031 |
| Rv1386 | PE15 | P0A682 |
| Rv1387 | PPE20 | Q7D8H6 |
| Rv1396c | PE_PGRS25 | P71664 |
| Rv1430 | PE16 | Q7D8G5 |
| Rv1441c | PE_PGRS26 | Q79FP3 |
| Rv1450c | PE_PGRS27 | Q79FP2 |
| Rv1452c | PE_PGRS28 | Q79FP1 |
| Rv1468c | PE_PGRS29 | Q79FP0 |
| Rv1548c | PPE21 | Q10778 |
| Rv1646 | PE17 | Q7D879 |
| Rv1651c | PE_PGRS30 | Q79FL8 |
| Rv1705c | PPE22 | Q79FL6 |
| Rv1706c | PPE23 | Q7D842 |
| Rv1753c | PPE24 | Q79FL2 |
| Rv1759c | wag22 | P0A686 |
| Rv1768 | PE_PGRS31 | Q79FK9 |
| Rv1787 | PPE25 | Q79FK8 |
| Rv1788 | PE18 | Q7D7Y9 |
| Rv1789 | PPE26 | Q79FK6 |
| Rv1790 | PPE27 | Q79FK5 |
| Rv1791 | PE19 | Q79FK4 |
| Rv1800 | PPE28 | Q79FK2 |
| Rv1801 | PPE29 | Q7D7X9 |
| Rv1802 | PPE30 | P0A692 |
| Rv1803c | PE_PGRS32 | Q79FJ9 |
| Rv1806 | PE20 | Q7D7X6 |
| Rv1807 | PPE31 | Q79FJ7 |
| Rv1808 | PPE32 | Q79FJ6 |
| Rv1809 | PPE33 | Q79FJ5 |
| Rv1818c | PE_PGRS33 | Q50615 |
| Rv1840c | PE_PGRS34 | Q50594 |
| Rv1917c | PPE34 | Q79FI9 |
| Rv1918c | PPE35 | Q79FI8 |
| Rv1983 | PE_PGRS35 | Q10873 |
| Rv2098c | PE_PGRS36 | P0A688 |
| Rv2099c | PE21 | Q79FH7 |
| Rv2107 | PE22 | Q7D7I5 |
| Rv2108 | PPE36 | P95315 |
| Rv2123 | PPE37 | Q79FH3 |
| Rv2162c | PE_PGRS38 | Q7D7F8 |
| Rv2328 | PE23 | P0A684 |
| Rv2340c | PE_PGRS39 | Q7D7A7 |
| Rv2352c | PPE38 | Q7D7A2 |
| Rv2356c | PPE40 | Q7D7A1 |
| Rv2371 | PE_PGRS40 | Q79FE9 |
| Rv2396 | PE_PGRS41 | Q79FE6 |
| Rv2408 | PE24 | Q79FE3 |
| Rv2430c | PPE41 | Q79FE1 |
| Rv2431c | PE25 | Q7D756 |
| Rv2487c | PE_PGRS42 | Q7D724 |
| Rv2490c | PE_PGRS43 | Q79FD4 |
| Rv2519 | PE26 | Q79FD3 |
| Rv2591 | PE_PGRS44 | Q50630 |
| Rv2608 | PPE42 | Q79FC6 |
| Rv2615c | PE_PGRS45 | Q79FC3 |
| Rv2634c | PE_PGRS46 | P0A690 |
| Rv2741 | PE_PGRS47 | Q79FB3 |
| Rv2768c | PPE43 | Q79FA9 |
| Rv2769c | PE27 | Q79FA8 |
| Rv2770c | PPE44 | Q79FA7 |
| Rv2853 | PE_PGRS48 | Q6MX26 |
| Rv2892c | PPE45 | P0A694 |
| Rv3018c | PPE46 | P31500 |
| Rv3022c | PPE48 | O53268 |
| Rv3097c | PE_PGRS63 | P77909 |
| Rv3125c | PPE49 | Q7D631 |
| Rv3135 | PPE50 | Q6MX07 |
| Rv3136 | PPE51 | Q7D623 |
| Rv3144c | PPE52 | Q6MX05 |
| Rv3159c | PPE53 | Q6MX04 |
| Rv3343c | PPE54 | Q6MWY2 |
| Rv3345c | PE_PGRS50 | Q6MWY0 |
| Rv3347c | PPE55 | Q6MWX9 |
| Rv3350c | PPE56 | Q6MWX8 |
| Rv3367 | PE_PGRS51 | Q6MWX7 |
| Rv3388 | PE_PGRS52 | Q6MWX5 |
| Rv3425 | PPE57 | Q50703 |
| Rv3426 | PPE58 | Q50702 |
| Rv3429 | PPE59 | O06246 |
| Rv3477 | PE31 | Q7D5G7 |
| Rv3478 | PPE60 | Q6MWX1 |
| Rv3507 | PE_PGRS53 | Q6MWW9 |
| Rv3508 | PE_PGRS54 | O53553 |
| Rv3511 | PE_PGRS55 | Q6MWW8 |
| Rv3514 | PE_PGRS57 | Q6MWW6 |
| Rv3532 | PPE61 | Q6MWW4 |
| Rv3533c | PPE62 | Q7D5C5 |
| Rv3539 | PPE63 | Q7D5B9 |
| Rv3558 | PPE64 | Q6MWW0 |
| Rv3590c | PE_PGRS58 | Q7D580 |
| Rv3595c | PE_PGRS59 | Q6MWV6 |
| Rv3621c | PPE65 | Q7D569 |
| Rv3622c | PE32 | Q7D568 |
| Rv3650 | PE33 | Q7D550 |
| Rv3652 | PE_PGRS60 | Q6MWV1 |
| Rv3738c | PPE66 | Q79FA3 |
| Rv3739c | PPE67 | Q79FA2 |
| Rv3812 | PE_PGRS62 | Q7D4U2 |
| Rv3872 | PE35 | Q79F93 |
| Rv3873 | PPE68 | Q79F92 |
| Rv3892c | PPE69 | Q79F90 |
